# Time-dependent propagators for stochastic models of gene expression: an analytical method

**DOI:** 10.1007/s00285-017-1196-4

**Published:** 2017-12-15

**Authors:** Frits Veerman, Carsten Marr, Nikola Popović

**Affiliations:** 1 School of Mathematics, University of Edinburgh, Edinburgh, UK; 2 Institute of Computational Biology, Helmholtz Zentrum München - German Research Center for Environmental Health, Ingolstädter Landstr. 1, 85764 Neuherberg, Germany

**Keywords:** Stochastic gene expression, Probability generating function, Propagator, Dynamical systems, Perturbation techniques, Asymptotic analysis, 34E05, 35C20, 35F40, 37N25, 60J25, 92C40

## Abstract

The inherent stochasticity of gene expression in the context of regulatory networks profoundly influences the dynamics of the involved species. Mathematically speaking, the propagators which describe the evolution of such networks in time are typically defined as solutions of the corresponding chemical master equation (CME). However, it is not possible in general to obtain exact solutions to the CME in closed form, which is due largely to its high dimensionality. In the present article, we propose an analytical method for the efficient approximation of these propagators. We illustrate our method on the basis of two categories of stochastic models for gene expression that have been discussed in the literature. The requisite procedure consists of three steps: a probability-generating function is introduced which transforms the CME into (a system of) partial differential equations (PDEs); application of the method of characteristics then yields (a system of) ordinary differential equations (ODEs) which can be solved using dynamical systems techniques, giving closed-form expressions for the generating function; finally, propagator probabilities can be reconstructed numerically from these expressions via the Cauchy integral formula. The resulting ‘library’ of propagators lends itself naturally to implementation in a Bayesian parameter inference scheme, and can be generalised systematically to related categories of stochastic models beyond the ones considered here.

## Introduction

### Motivation

**Fig. 1:**
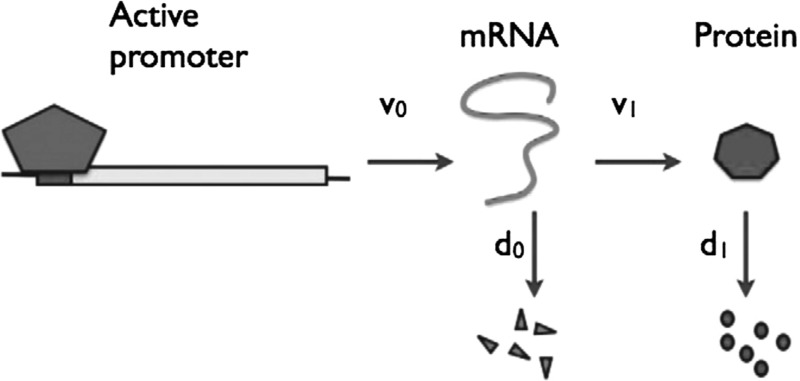
The canonical model of gene expression. Transcription of mRNA occurs with rate $\nu _0$; mRNA is translated to protein with rate $\nu _1$. Both mRNA and protein decay, with rates $d_0$ and $d_1$, respectively. Figure courtesy of Shahrezaei and Swain (2008a) (Copyright (2008) National Academy of Sciences, U.S.A.)

Understanding the process of gene expression in the context of gene regulatory networks is indispensable for gaining insight into the fundamentals of numerous biological processes. However, gene expression can be highly stochastic in nature, both in prokaryotic and in eukaryotic organisms; see e.g. the work by Elowitz et al. (2002), Raj and Oudenaarden (2008), Shahrezaei and Swain (2008b), and the references therein. This inherent stochasticity has a profound influence on the dynamics of the involved species, in particular when their abundance is low. Therefore, gene expression is often appropriately described by stochastic models (Bressloff 2014; Karlebach and Shamir 2008; Thattai and Oudenaarden 2001; Wilkinson 2009). A schematic of the canonical model for gene expression is depicted in Fig. 1. Here, the processes of transcription, translation, and degradation are approximated by single rates.

**Fig. 2:**
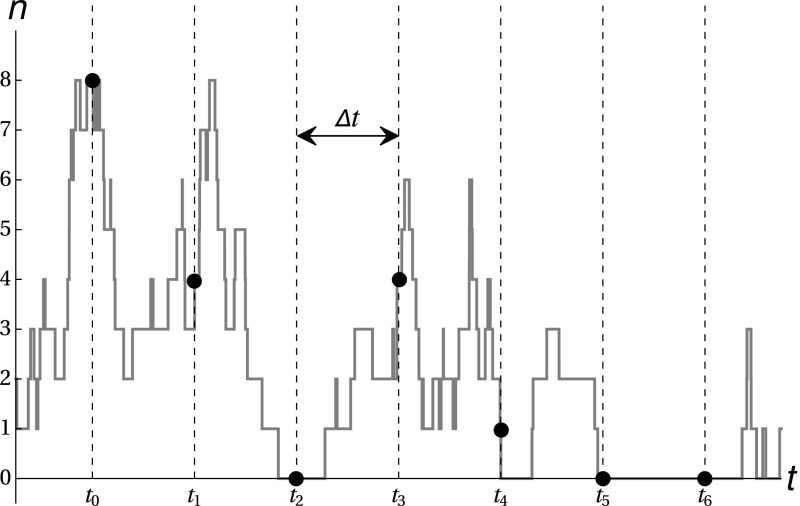
Sketch of a potential time series of protein abundance *n*, sampled at times $t_i$, with regular, fixed, sampling interval $\Delta t$

To test the validity of such stochastic models, a comparison with experimental data needs to be performed. The development of experimental techniques, such as time-lapse fluorescence microscopy (Coutu and Schroeder 2013; Elowitz et al. 2002; Larson et al. 2011; Muzzey and Oudenaarden 2009; Raj et al. 2006; Young et al. 2011), allows for real-time tracking of gene expression dynamics in single cells, providing mRNA or protein abundance time series, such as those depicted in Fig. 2. To select between competing hypotheses on the underlying regulatory networks given measurement data, as well as to infer the values of the corresponding model parameters, we can apply Bayesian inference theory to calculate the likelihood of a given model, which is constructed as follows.

The abundance of a protein, denoted by *n*, is sampled at times $t_i$, see Fig. 2, yielding a list of measurement pairs $(t_i,n_i)$ from which *transitions* $(\Delta t,n_i \rightarrow n_{i+1})$ between states can be extracted; here, $\Delta t = t_{i+1}-t_i$ is the regular, fixed, sampling interval. Next, the underlying stochastic model with parameter set $\Theta$ is used to calculate the probabilities of these transitions, which are denoted by $P_{n_{i+1} | n_i}(\Delta t)$. These so-called *propagators* give the probability of $n_{i+1}$ protein being present after time $\Delta t$, given an initial protein abundance of $n_i$. The log-likelihood $L(\Theta |D)$ of the parameter set $\Theta$, given the observed data *D*, is now defined in terms of the propagators as

1.1 $$\begin{aligned} L(\Theta |D) = \sum _i \log \,P_{n_{i+1} | n_i}(\Delta t). \end{aligned}$$

To infer the values of parameters in the model, propagators are calculated for a wide range of parameter combinations, resulting in a ‘log-likelihood landscape’; the maximal value of the log-likelihood as a function of the model parameters yields the most likely parameter values, given the experimental data. An example realisation of the above procedure can e.g. be found in the work by Feigelman et al. (2015).

To calculate accurately the log-likelihood in (1.1), it is imperative that the values of the propagators can be extracted from the underlying stochastic model for any desired combination of parameters in $\Theta$. In particular, we need to be able to calculate the propagator $P_{n|n_0}(t)$ as a function of time *t* for any initial protein number $n_0$. Unfortunately, the highly complex nature of the stochastic models involved makes it very difficult to obtain explicit expressions for these probabilities. Some analytical progress can be made when a steady-state approximation is performed, i.e. when it is assumed that the system is allowed to evolve for a sufficiently long time, such that it converges to a time-independent state. However, the sampling interval $\Delta t$ used for obtaining experimental data, as seen in Fig. 2, is often *short* with respect to the protein life time. As that life time represents a natural time scale for the system dynamics, it follows that the evolution of the probabilities $P_{n|n_0}(t)$ should be studied over short times, in contradiction with the steady-state or long-evolution-time approximations which have previously been employed to derive analytical results (Bokes et al. 2012a; Hornos et al. 2005; Iyer-Biswas and Jayaprakash 2014; Shahrezaei and Swain 2008a).

The complex nature of stochastic models for gene expression has led to the widespread use of stochastic simulation techniques, such as Gillespie’s algorithm (Gillespie 1977), with the aim of predicting values for the associated propagators from these models; see Feigelman et al. (2016) for recent work combining stochastic simulation with a particle filtering approach. However, these approaches can still be very time-consuming, due to the (relatively) high dimensionality of the model parameter space, combined with the fact that, for each combination of parameter values, the stochastic model has to be simulated sufficiently many times to yield a probability distribution that can be used to infer the corresponding propagator. For that reason, it is desirable to be able to obtain explicit expressions for the propagator $P_{n|n_0}(t)$ directly in terms of the model parameters, if necessary in an appropriate approximation.

### Analytical method

In the present article, we develop an analytical method for the efficient evaluation of time-dependent propagators in stochastic gene expression models, for arbitrary values of the model parameters. The results of our analysis can be implemented in a straightforward fashion in a Bayesian parameter inference framework, as outlined above.

**Fig. 3:**
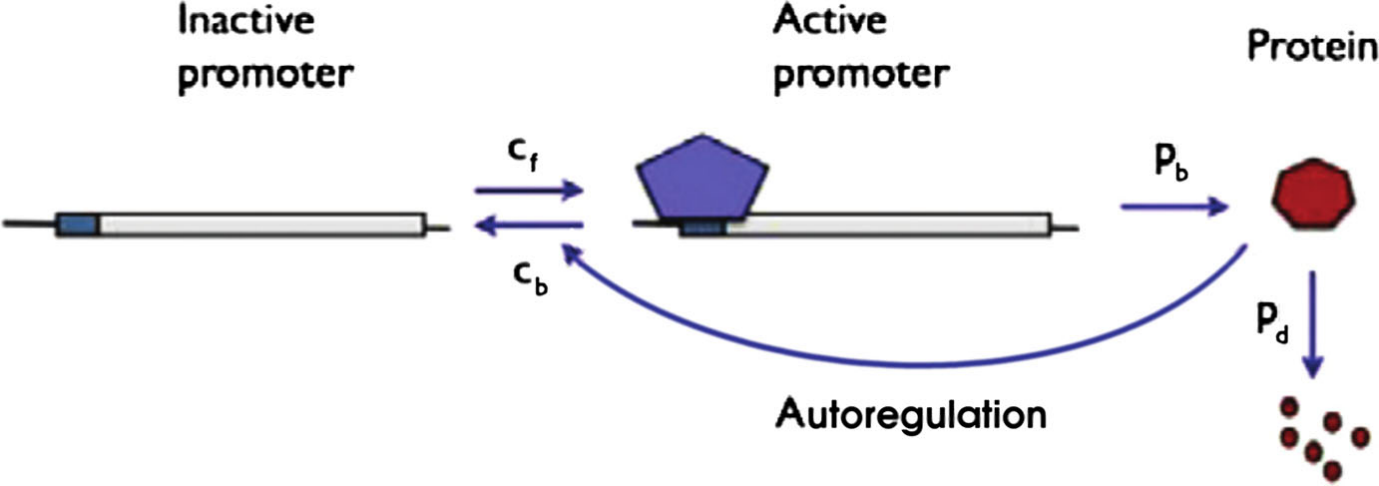
Schematic of model A, a gene expression model with autoregulation. Base figure courtesy of Shahrezaei and Swain (2008a). (Copyright (2008) National Academy of Sciences, U.S.A.)

**Fig. 4:**
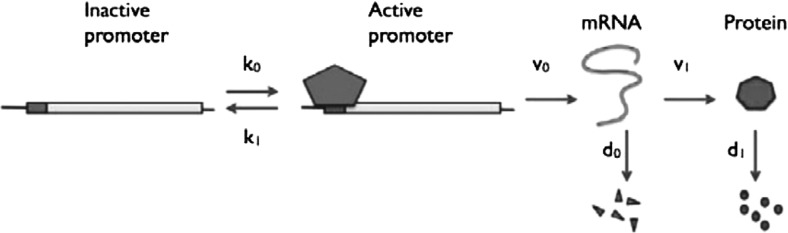
Schematic of model B, a gene expression model that explicitly incorporates transcription (Shahrezaei and Swain 2008a). Figure courtesy of Shahrezaei and Swain (2008a). (Copyright (2008) National Academy of Sciences, U.S.A.)

To demonstrate our approach, we analyse two different stochastic models for gene expression. The first model, henceforth referred to as ‘model A’, is a model that incorporates autoregulation, where transcription and translation are approximated by single rates and protein can either stimulate or inhibit its own production by influencing the activity of DNA; see Fig. 3. That model was first studied by Iyer-Biswas and Jayaprakash (2014) via a steady-state approximation. The second model, henceforth referred to as ‘model B’, models both mRNA and protein explicitly and again incorporates DNA switching between an active and an inactive state; see Fig. 4. That model was first studied by Shahrezaei and Swain (2008a) in a long-evolution-time approximation.

Both model A and model B are formulated in terms of the chemical master equation (CME), which is the accepted mathematical representation of stochastic gene expression in the context of the model categories considered here; cf. Iyer-Biswas and Jayaprakash (2014) and Shahrezaei and Swain (2008a), respectively. Mathematically speaking, the CME is an infinite-dimensional system of linear ordinary differential equations (ODEs) that describes the evolution in time of the probabilities of observing a specific state in the system, given some initial state. Numerous approaches have been suggested for the (approximate) solution of the CME; see e.g. Popović et al. (2016) and the references therein for details. Our method relies on a combination of various techniques from the theory of differential equations and dynamical systems; specifically, we perform three consecutive steps, as follows.

1. **CME system** $\rightarrow$ **PDE system:** We introduce a probability-generating function to convert the CME into a (system of) partial differential equations (PDEs).
2. **PDE system** $\rightarrow$ **ODE system:** Applying the method of characteristics—combined, if necessary, with perturbation techniques—we transform the system of PDEs obtained in step 1 into a dynamical system, that is, a system of ODEs.
3. **ODE system** $\rightarrow$ **Explicit solution:** Making use of either special functions (model A) or multiple-time-scale analysis (model B), we obtain explicit solutions to the dynamical system found in step 2.

We emphasise that the ‘characteristic system’ of ODEs which is obtained in step 2 is low-dimensional, in contrast to the underlying CME system, as well as that it exhibits additional structure, allowing for the derivation of a closed-form analytical approximation for the associated generating function.

To convert the results of the above procedure into solutions to the original stochastic model, the three steps involved in our analysis have to be reverted. To that end, we require the following three ingredients:

1. Initial conditions are originally stated in terms of the CME, and first have to be reformulated in terms of the corresponding system of PDEs to ensure well-posedness; then, initial conditions can be extracted for the dynamical system that was obtained via the method of characteristics, reverting step 3.
2. To transform solutions to the characteristic system into solutions of the underlying PDE system, the associated ‘characteristic transformation’ has to be inverted, reverting step 2.
3. Lastly, solutions of the CME have to be extracted from solutions to the resulting PDE system, reverting step 1. Although the correspondence between the two sets of solutions is exact, theoretically speaking, the complexity of the expressions involved precludes the efficient analytical reconstruction of propagators from their generating functions. Therefore, we propose a novel hybrid analytical-numerical approach which relies on the Cauchy integral formula.

**Fig. 5:**
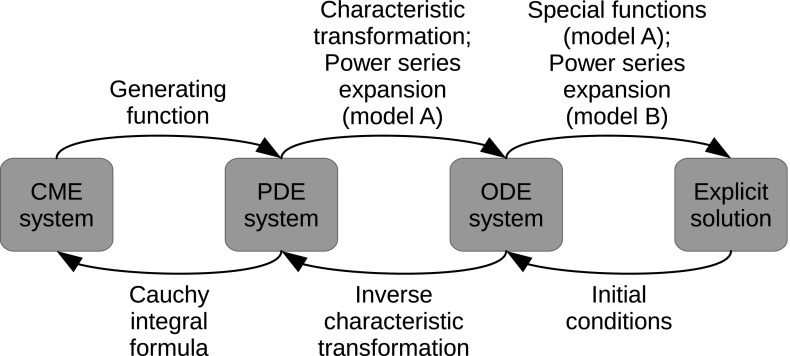
Schematic overview of the analytical method

The various steps in our analytical method, as indicated above, are represented in Fig. 5. It is important to mention that the implementation of Bayesian parameter inference, as outlined in Sect. 1.1, is not a topic for the present article; rather, the aim here is to describe our method, and to present analytical results which can readily be implemented in the context of parameter inference. The article hence realises the first stage of our research programme; the natural next stage, which is precisely that implementation, will be the subject of a follow-up article by the same authors.

### Outline

The present article is organised as follows. In Sect. 2, we apply the analytical method outlined in Sect. 1.2 to model A, the gene expression model with autoregulation. Here, we use a perturbative approach to incorporate the autoregulatory aspects of the model; the resulting dynamical system can be solved in terms of confluent hypergeometric functions, see §13 in NIST Digital Library of Mathematical Functions . In Sect. 3, the same method is applied to model B, the model that explicitly incorporates transcription. We also indicate how autoregulation can be added to that model, and how the resulting extended model can be analysed on the basis of our treatment of model A. The analysis carried out in Sects. 2 and 3 yields a ‘library’ of explicit asymptotic expressions for the probability-generating functions associated to the underlying stochastic models. To obtain quantifiable expressions for their propagators, we introduce a novel hybrid analytical–numerical approach in Sect. 4, which can be readily implemented in the Bayesian parameter inference framework that provided the motivation for our analysis; see Sect. 1.1. We conclude with a discussion of our results, and an outlook to future work, in Sect. 5.

## Model A: gene expression with autoregulation

We first demonstrate our analytical method in the context of an autoregulatory stochastic gene expression model, as presented by Iyer-Biswas and Jayaprakash (2014); see also Fig. 3. In the original article (Iyer-Biswas and Jayaprakash 2014), a Poisson representation was used to obtain analytical descriptions for time-independent solutions to the model. For a visual guide to the upcoming analysis, the reader is referred to Fig. 5.

### Stochastic model and CME

The basic stochastic model for gene expression is represented by the reaction scheme2.1The gene can hence switch between the inactive state *D* and the active state $D^*$, with switching rates $c_f$ and $c_b$, respectively. The active gene produces protein (*P*) with rate $p_b$, while protein decays with rate $p_d$.

The autoregulatory part of the model is implemented as either positive or negative feedback:

2.2a $$\begin{aligned}&\phantom {D^* + } D + P \quad {\mathop {\rightarrow }\limits ^{a}} \quad D^* + P \phantom {+ D} \quad \text {(autoactivation)}, \end{aligned}$$

2.2b $$\begin{aligned}&\phantom {D + } D^* + P \quad {\mathop {\rightarrow }\limits ^{r}} \quad D + P \phantom {+ D^*}\quad \text {(autorepression)}. \end{aligned}$$

In the case of autoactivation, viz. (2.2a), protein induces activation of the gene with activation rate *a*, thereby accelerating its own production; in the case of autorepression, viz. (2.2b), protein deactivates the active gene with repression rate *r*, impeding its own production.

The CME system that is associated to the reaction scheme in (2.1), with autoactivation as in (2.2a), is given by

2.3a $$\begin{aligned} \frac{\text {d} P^{(0)}_n}{\text {d} t}&= -\left( \kappa _f + \frac{a}{p_d} n\right) P^{(0)}_n + \kappa _b P^{(1)}_n + \left[ (n+1) P^{(0)}_{n+1} - n P^{(0)}_n \right] , \end{aligned}$$

2.3b $$\begin{aligned} \frac{\text {d} P^{(1)}_n}{\text {d} t}&= \left( \kappa _f + \frac{a}{p_d} n\right) P^{(0)}_n {-} \kappa _b P^{(1)}_n {+} \left[ (n+1) P^{(1)}_{n+1} {-} n P^{(1)}_n \right] + \lambda \left[ P^{(1)}_{n-1} - P^{(1)}_n\right] . \end{aligned}$$

Here, $P^{(j)}_n(t)$ ($j=0,1$) represents the probability of *n* protein being present at time *t* while the gene is either inactive (0) or active (1). The time variable is nondimensionalised by the protein decay rate $p_d$; other model parameters are scaled as

2.4 $$\begin{aligned} \kappa _f = \frac{c_f}{p_d},\quad \kappa _b = \frac{c_b}{p_d},\quad \text {and}\quad \lambda = \frac{p_b}{p_d}. \end{aligned}$$

Analogously, the CME system for the case of autorepression, as defined in (2.2b), is given by

2.5a $$\begin{aligned} \frac{\text {d} P^{(0)}_n}{\text {d} t}&= -\kappa _f P^{(0)}_n + \left( \kappa _b + \frac{r}{p_d} n\right) P^{(1)}_n + \left[ (n+1) P^{(0)}_{n+1} - n P^{(0)}_n \right] , \end{aligned}$$

2.5b $$\begin{aligned} \frac{\text {d} P^{(1)}_n}{\text {d} t}&= \kappa _f P^{(0)}_n {-} \left( \kappa _b {+} \frac{r}{p_d} n\right) P^{(1)}_n {+} \left[ (n+1) P^{(1)}_{n+1} {-} n P^{(1)}_n \right] {+} \lambda \left( P^{(1)}_{n-1} - P^{(1)}_n\right) . \end{aligned}$$

#### Remark 2.1

A priori, it is possible to incorporate both autoactivation and autorepression in a single model, by merging systems (2.3) and (2.5). However, since autoactivation and autorepression precisely counteract each other, a partial cancellation would ensue, resulting in *effective* activation or repression. It can hence be argued that the simultaneous inclusion of both effects would introduce superfluous terms and parameters, which could be considered as poor modelling practice. Therefore, we choose to model the two autoregulation mechanisms separately.

### Generating function PDE

Rather than investigating the dynamics of (2.3) and (2.5) numerically, using stochastic simulation, we aim to employ an analytical approach. To that end, we define the probability-generating functions $F^{(j)}(z,t)$ ($j=0,1$) as follows; see e.g. Gardiner (2009):

2.6 $$\begin{aligned} F^{(j)}(z,t) = \sum _{n=0}^\infty z^n P^{(j)}_n(t). \end{aligned}$$

In the case of autoactivation, the generating functions $F^{(j)}(z,t)$ can be seen to satisfy

2.7a $$\begin{aligned} \partial _t F^{(0)} + (z-1) \partial _z F^{(0)} + \frac{a}{p_d} z \partial _z F^{(0)}&= -\kappa _f F^{(0)} + \kappa _b F^{(1)}, \end{aligned}$$

2.7b $$\begin{aligned} \partial _t F^{(1)} + (z-1) \partial _z F^{(1)} - \frac{a}{p_d} z \partial _z F^{(0)}&= \kappa _f F^{(0)} - \kappa _b F^{(1)} + \lambda (z-1) F^{(1)} \end{aligned}$$

if the coefficients $P^{(j)}_n(t)$ in (2.6) obey the CME system (2.3); likewise, in the case of autorepression, (2.3) gives rise to

2.8a $$\begin{aligned} \partial _t F^{(0)} + (z-1) \partial _z F^{(0)} - \frac{r}{p_d} z \partial _z F^{(1)}&= -\kappa _f F^{(0)} + \kappa _b F^{(1)}, \end{aligned}$$

2.8b $$\begin{aligned} \partial _t F^{(1)} + (z-1) \partial _z F^{(1)} + \frac{r}{p_d} z \partial _z F^{(1)}&= \kappa _f F^{(0)} - \kappa _b F^{(1)} + \lambda (z-1) F^{(1)}. \end{aligned}$$

Both (2.7) and (2.8) are systems of coupled, linear, first-order, hyperbolic partial differential equations (PDEs). Systems of this type are typically difficult to analyse; existing techniques only provide general results (Courant and Hilbert 1962; Taylor 2011).

To allow for an explicit analysis of systems (2.7) and (2.8), we make the following assumption:

#### Assumption 2.2

We assume that the autoactivation rate *a* in (2.2) is *small* in comparison with the other model parameters; specifically, we write

2.9 $$\begin{aligned} a = \alpha p_d\,\delta , \end{aligned}$$

where $0<\delta <1$ is sufficiently small. Likewise, we assume that the autorepression rate *r* is small in comparison with the other model parameters, writing

2.10 $$\begin{aligned} r = \rho p_d\,\delta . \end{aligned}$$

Previous work on the inclusion of autoregulatory effects in model selection by Feigelman et al. (2016) suggests that, in the context of Nanog expression in mouse embryonic stem cells, autoregulation rates are indeed small compared to other model parameters.

Based on Assumption 2.2, we can expand the generating functions $F^{(j)}$ ($j=0,1$) as power series in $\delta$:

2.11 $$\begin{aligned} F^{(j)}(z,t) = \sum _{m=0}^\infty \delta ^m F^{(j)}_m(z,t). \end{aligned}$$

Substitution of (2.11) into (2.7) yields

2.12a $$\begin{aligned} \partial _t F^{(0)}_m + (z-1) \partial _z F^{(0)}_m&= -\kappa _f F^{(0)}_m + \kappa _b F^{(1)}_m - \alpha \, z \partial _z F^{(0)}_{m-1}, \end{aligned}$$

2.12b $$\begin{aligned} \partial _t F^{(1)}_m + (z-1) \partial _z F^{(1)}_m&= \kappa _f F^{(0)}_m - \kappa _b F^{(1)}_m + \lambda (z-1) F^{(1)}_m + \alpha \, z \partial _z F^{(0)}_{m-1};\nonumber \\ \end{aligned}$$

analogously, we substitute (2.11) into (2.8) to find

2.13a $$\begin{aligned} \partial _t F^{(0)}_m + (z-1) \partial _z F^{(0)}_m&= -\kappa _f F^{(0)}_m + \kappa _b F^{(1)}_m + \rho \, z \partial _z F^{(1)}_{m-1}, \end{aligned}$$

2.13b $$\begin{aligned} \partial _t F^{(1)}_m + (z-1) \partial _z F^{(1)}_m&= \kappa _f F^{(0)}_m - \kappa _b F^{(1)}_m + \lambda (z-1) F^{(1)}_m - \rho \, z \partial _z F^{(1)}_{m-1}.\nonumber \\ \end{aligned}$$

We observe that, in (2.12) and (2.13), the same leading-order differential operator acts on both $F^{(0)}_m$ and $F^{(1)}_m$, which allows us to apply the method of characteristics to solve the equations for $F^{(j)}_m$ ($j=0,1$) *simultaneously*. In particular, we emphasise that, mathematically speaking, the resulting perturbation is *regular* in the perturbation parameter $\delta$.

### Dynamical systems analysis

In this section, we apply the method of characteristics to derive the ‘characteristic equations’ that are associated to the PDE systems (2.12) and (2.13), respectively; the former are systems of ODEs, which are naturally analysed in the language of dynamical systems.

#### Autoactivation

We first consider the case of autoactivation; to that end, we rewrite system (2.12) as

2.14a $$\begin{aligned} \big (\partial _t + v \partial _v\big ) F^{(0)}_m+ \kappa _f F^{(0)}_m - \kappa _b F^{(1)}_m&= - \alpha (v+1) \partial _v F^{(0)}_{m-1}, \end{aligned}$$

2.14b $$\begin{aligned} \big (\partial _t + v \partial _v\big ) F^{(1)}_m- \kappa _f F^{(0)}_m + \kappa _b F^{(1)}_m - \lambda v F^{(1)}_m&= \alpha \, (v+1) \partial _v F^{(0)}_{m-1}, \end{aligned}$$

where we have introduced the new variable

2.15 $$\begin{aligned} v = z-1. \end{aligned}$$

The differential operator $\partial _t + v \partial _v$ in Eq. (2.14) gives rise to characteristics $\xi (s;v_0)$ that obey the characteristic equation

2.16 $$\begin{aligned} \frac{\partial v}{\partial s} = v; \end{aligned}$$

these characteristics can thus be expressed as

2.17 $$\begin{aligned} \xi (s;v_0) = \left( s, v_0\mathrm{e}^{s}\right) . \end{aligned}$$

Since the partial differential operators in (2.14) transform into

2.18a $$\begin{aligned} \partial _t + v \partial _v&= \partial _s\quad \text {and} \end{aligned}$$

2.18b $$\begin{aligned} \alpha (v+1) \partial _v&= \alpha \left( v_0 +\mathrm{e}^{-s}\right) \partial _{v_0}, \end{aligned}$$

we arrive at the following system:

2.19a $$\begin{aligned} \partial _s F^{(0)}_m + \kappa _f F^{(0)}_m - \kappa _b F^{(1)}_m&= - \alpha \left( v_0 + \mathrm{e}^{-s}\right) \partial _{v_0} F^{(0)}_{m-1}, \end{aligned}$$

2.19b $$\begin{aligned} \partial _s F^{(1)}_m - \kappa _f F^{(0)}_m + \kappa _b F^{(1)}_m - \lambda v_0 \mathrm{e}^{s} F^{(1)}_m&= \alpha \left( v_0 + \mathrm{e}^{-s}\right) \partial _{v_0} F^{(0)}_{m-1}. \end{aligned}$$

Note that Eq. (2.19) is a recursive (nonhomogeneous) system of *ordinary* differential equations for $F^{(j)}_m$ ($j=0,1$). Henceforth, we will therefore refer to (2.19) as such, while retaining the use of partial derivatives $\partial _s$ due to the presence of $\partial _{v_0}$ in the corresponding right-hand sides.

To solve system (2.19), we rewrite it as a second-order ODE for $F^{(0)}_m$: we hence obtain

2.20 $$\begin{aligned} \Big [\partial _s^2 + \big (\kappa _f + \kappa _b - \lambda v_0 \mathrm{e}^s\big ) \partial _s - \kappa _f \lambda v_0 \mathrm{e}^s \Big ] F^{(0)}_m = \big [\lambda v_0 \mathrm{e}^s -\partial _s\big ]\,\alpha \big (v_0 + \mathrm{e}^{-s}\big ) \partial _{v_0} F^{(0)}_{m-1}, \end{aligned}$$

which can be solved recursively to determine $F^{(0)}_m$ for any $m\ge 0$. To simplify (2.20), we introduce the variable

2.21 $$\begin{aligned} w = \lambda v_0\mathrm{e}^{s}, \end{aligned}$$

which transforms the partial derivatives $\partial _s$ and $\partial _{v_0}$ into

2.22 $$\begin{aligned} \partial _s \rightarrow w \partial _w\qquad \text {and}\qquad \partial _{v_0} \rightarrow \frac{w}{v_0}\partial _w + \partial _{v_0}; \end{aligned}$$

Equation (2.20) hence reads

2.23 $$\begin{aligned}&\Big [(w\partial _w)^2 + (\kappa _f + \kappa _b - w) (w\partial _w) - \kappa _f w \Big ] F^{(0)}_m \nonumber \\&\quad = \big (w -w\partial _w\big )\,\alpha \left( w+ \lambda \right) \left( \partial _w + \frac{v_0}{w}\partial _{v_0}\right) F^{(0)}_{m-1}. \end{aligned}$$

Using (2.19a), we can express the second component $F^{(1)}_m$ in terms of $F^{(0)}_m$ as

2.24 $$\begin{aligned} F^{(1)}_m&= \frac{1}{\kappa _b}\left[ \partial _s F^{(0)}_m + \kappa _f F^{(0)}_m + \alpha \left( v_0 + \mathrm{e}^{-s}\right) \partial _{v_0} F^{(0)}_{m-1}\right] \nonumber \\&= \frac{1}{\kappa _b}\left[ w \partial _w F^{(0)}_m + \kappa _f F^{(0)}_m + \alpha (w+\lambda )\left( \partial _w + \frac{v_0}{w}\partial _{v_0}\right) F^{(0)}_{m-1}\right] . \end{aligned}$$

At leading order, i.e. for $m=0$, (2.23) reduces to

2.25 $$\begin{aligned} \big [(w \partial _w)^2 + (\kappa _f + \kappa _b - w)(w \partial _w) - \kappa _f w\big ] F^{(0)}_0 = 0, \end{aligned}$$

the solutions of which can be expressed in terms of the confluent hypergeometric function ${}_1 F_1$, see §13 of NIST Digital Library of Mathematical Functions , to yield

2.26 $$\begin{aligned} F^{(0)}_0(w) = c_1 {}_1 F_1(\kappa _f,1+\kappa _f+\kappa _b,w)+ c_2 w^{-\kappa _f-\kappa _b} {}_1 F_1 (-\kappa _b,1-\kappa _f-\kappa _b,w). \end{aligned}$$

Using (2.24), we can determine

2.27 $$\begin{aligned} F^{(1)}_0(w) = \frac{1}{\kappa _b}\left( w\partial _w F^{(0)}_0 + \kappa _f F^{(0)}_0\right) , \end{aligned}$$

with $F^{(0)}_0$ as given in (2.26); for an explicit expression, see Eq. (A.1) in Appendix A.

The expression for $F^{(0)}_0(w)$ in (2.26) allows us to determine the first-order correction $F^{(0)}_1(w)$: substituting $m=1$ in (2.23), we obtain

2.28 $$\begin{aligned}&\Big [(w\partial _w)^2 + (\kappa _f + \kappa _b - w) (w\partial _w) - \kappa _f w\Big ] F^{(0)}_1 \nonumber \\&\quad = \big (w -w\partial _w\big )\,\alpha \left( w+ \lambda \right) \left( \partial _w + \frac{v_0}{w}\partial _{v_0}\right) F^{(0)}_0. \end{aligned}$$

Next, we apply the method of variation of constants to express the solution to (2.28) as

2.29 $$\begin{aligned} F^{(0)}_1(w)&=\frac{1}{\kappa _f+\kappa _b} \Bigg [{}_1 F_1 (\kappa _f,1+\kappa _f+\kappa _b,w) \int _{c_3}^w \!\!\!{}_1 F_1({-}\kappa _b,1{-}\kappa _f{-}\kappa _b,\hat{w}) \, g(\hat{w}) \;\text {d} \hat{w}\nonumber \\&\quad -w^{-\kappa _f-\kappa _b} {}_1 F_1 (-\kappa _b,1-\kappa _f-\kappa _b,w) \int _{c_4}^w \!\!\!{}_1 F_1(\kappa _f,1+\kappa _f\nonumber \\&\quad +\kappa _b,\hat{w})\, \frac{g(\hat{w})}{\hat{w}^{-\kappa _f-\kappa _b}} \;\text {d} \hat{w}\Bigg ], \end{aligned}$$

where

2.30 $$\begin{aligned} g(w) = \frac{\mathrm{e}^{-w}}{w}(w -w\partial _w)\,\alpha \left( w+ \lambda \right) \left( \partial _w + \frac{v_0}{w}\partial _{v_0}\right) F^{(0)}_0, \end{aligned}$$

with $F^{(0)}_0$ as given in (2.26). Finally, we may again use (2.24) to determine

2.31 $$\begin{aligned} F^{(1)}_1(w) = \frac{1}{\kappa _b}\left[ w\partial _w F^{(0)}_1 + \kappa _f F^{(0)}_1 + \alpha (w+\lambda )\left( \partial _w + \frac{v_0}{w}\partial _{v_0}\right) F^{(0)}_0\right] , \end{aligned}$$

with $F^{(0)}_0$ and $F^{(0)}_1$ as given in (2.26) and (2.29), respectively.

At this point in the analysis, the constants $c_{1}$ and $c_{2}$ in (2.26) and the integration limits $c_{3}$ and $c_{4}$ in (2.29) remain undetermined. To fix these constants, and thereby determine a unique solution to (2.19), we have to prescribe appropriate initial conditions.

#### Autorepression

Given the analysis of autoactivation in the previous subsection, the case of autorepression can be analysed in an analogous manner. Employing the same characteristics as before, recall (2.17), we obtain

2.32a $$\begin{aligned} \partial _s F^{(0)}_m+ \kappa _f F^{(0)}_m - \kappa _b F^{(1)}_m&= \rho \left( v_0 + \mathrm{e}^{-s}\right) \partial _{v_0} F^{(1)}_{m-1}, \end{aligned}$$

2.32b $$\begin{aligned} \partial _s F^{(1)}_m- \kappa _f F^{(0)}_m + \kappa _b F^{(1)}_m - \lambda v_0 \mathrm{e}^{s} F^{(1)}_m&= -\rho \left( v_0 + \mathrm{e}^{-s}\right) \partial _{v_0} F^{(1)}_{m-1} \end{aligned}$$

from system (2.13); cf. Eq. (2.19). Next, we rewrite (2.32) as a second-order ODE for $F^{(1)}_m$, using again the variable transformation in (2.21):

2.33 $$\begin{aligned}&\left[ \left( w \partial _w\right) ^2 + \left( \kappa _f + \kappa _b - w\right) \left( w \partial _w\right) - (1+\kappa _f) w \right] F^{(1)}_m \nonumber \\&\quad = - \left( w\partial _w\right) \rho \left( w + \lambda \right) \left( \partial _w + \frac{v_0}{w}\partial _{v_0}\right) F^{(1)}_{m-1}, \end{aligned}$$

which can be solved recursively to obtain $F^{(1)}_m$ for any $m\ge 1$. The first component $F^{(0)}_m$ can be expressed in terms of $F^{(1)}_m$ as

2.34 $$\begin{aligned} F^{(0)}_m = \frac{1}{\kappa _f} \left[ w\partial _w F^{(1)}_m + (\kappa _b - w) F^{(1)}_m + \rho \left( w + \lambda \right) \left( \partial _w + \frac{v_0}{w}\partial _{v_0}\right) F^{(1)}_{m-1}\right] ; \end{aligned}$$

to leading order, we thus obtain

2.35 $$\begin{aligned} F^{(1)}_0(w) = \hat{c}_1\,{}_1 F_1(1+\kappa _f,1+\kappa _f+\kappa _b,w) + \hat{c}_2 w^{-\kappa _f-\kappa _b}{}_1 F_1(1-\kappa _b,1-\kappa _f-\kappa _b,w) \end{aligned}$$

and

2.36 $$\begin{aligned} F^{(0)}_0 = \frac{1}{\kappa _f}\left[ w\partial _w F^{(1)}_0 + (\kappa _b - w) F^{(1)}_0 \right] . \end{aligned}$$

The corresponding equation for the first-order correction $F^{(1)}_1$ reads

2.37 $$\begin{aligned}&\Big [(w \partial _w)^2 + (\kappa _f + \kappa _b - w) (w \partial _w) - (1+\kappa _f) w\Big ] F^{(1)}_1 \nonumber \\&\quad = - (w\partial _w) \rho (w + \lambda )\left( \partial _w + \frac{v_0}{w}\partial _{v_0}\right) F^{(1)}_0, \end{aligned}$$

which can be solved via the method of variation of constants to give

2.38 $$\begin{aligned} F^{(1)}_1(w)&=\frac{1}{\kappa _f+\kappa _b} \Bigg [{}_1 F_1 (1+\kappa _f,1+\kappa _f+\kappa _b,w)\nonumber \\&\quad \int _{\hat{c}_3}^w \!\!\!{}_1 F_1(1-\kappa _b,1-\kappa _f-\kappa _b,\hat{w}) \, h(\hat{w}) \;\text {d} \hat{w}\nonumber \\&\quad -w^{-\kappa _f-\kappa _b} {}_1 F_1 (1-\kappa _b,1-\kappa _f-\kappa _b,w) \nonumber \\&\quad \int _{\hat{c}_4}^w \!\!\!{}_1 F_1(1+\kappa _f,1+\kappa _f +\kappa _b,\hat{w})\, \frac{h(\hat{w})}{\hat{w}^{-\kappa _f-\kappa _b}} \;\text {d} \hat{w}\Bigg ]; \end{aligned}$$

here,

2.39 $$\begin{aligned} h(w) = -\frac{\mathrm{e}^{-w}}{w} \left( w\partial _w\right) \rho \left( w + \lambda \right) \left( \partial _w + \frac{v_0}{w}\partial _{v_0}\right) F^{(1)}_0, \end{aligned}$$

with $F^{(1)}_0$ as in (2.35). The first-order correction to the first component $F^{(0)}_0$ is hence given by

2.40 $$\begin{aligned} F^{(0)}_1(w) = \frac{1}{\kappa _f} \left[ w\partial _w F^{(1)}_1 + (\kappa _b - w) F^{(1)}_1 + \rho \left( w + \lambda \right) \left( \partial _w + \frac{v_0}{w}\partial _{v_0}\right) F^{(1)}_0\right] , \end{aligned}$$

with $F^{(1)}_0$ and $F^{(1)}_1$ as in (2.35) and (2.38), respectively. As in the case of autoactivation, the constants $\hat{c}_{1}$ and $\hat{c}_2$ in (2.35) and the integration limits $\hat{c}_{3}$ and $\hat{c}_4$ in (2.38) remain undetermined, and have to be fixed through suitable initial conditions.

### Initial conditions

To determine appropriate initial conditions for the dynamical systems (2.19) and (2.32), we consider the original CME systems (2.3) and (2.5), respectively.

At time $t=0$, we impose an initial protein number $n=n_0$, which implies

2.41 $$\begin{aligned} P^{(0)}_n(0) + P^{(1)}_n(0) = \delta _{n,n_0} \end{aligned}$$

for the probabilities $P^{(j)}_n(t)$ ($j=0,1$); here, $\delta _{n,n_0}$ denotes the standard Kronecker symbol, with $\delta _{n,n_0}=1$ for $n=n_0$ and $\delta _{n,n_0}=0$ otherwise. Using the definition of the generating functions $F^{(j)}(z,t)$ in (2.6), we find that

2.42 $$\begin{aligned} F^{(0)}(z,0) + F^{(1)}(z,0) = z^{n_0} = (v+1)^{n_0}, \end{aligned}$$

taking into account the change of variables in (2.15). Thus, (2.42) provides an initial condition for the PDE systems (2.7) and (2.8). Given the power series expansion in (2.11), we infer that the coefficients $F^{(j)}_m(v,t)$ ($j=0,1$) satisfy

2.43a $$\begin{aligned} F^{(0)}_0(v,0) + F^{(1)}_0(v,0)&= (v+1)^{n_0} \quad \text {and} \end{aligned}$$

2.43b $$\begin{aligned} F^{(0)}_m(v,0) + F^{(1)}_m(v,0)&= 0 \quad \text {for all } m\ge 1, \end{aligned}$$

which holds for both (2.12) and (2.13).

To be able to interpret the initial conditions in (2.43) in the context of the dynamical systems (2.19) and (2.32), we revisit the method of characteristics, which was used to map the PDE systems (2.12) and (2.13) to the former, respectively.

**Fig. 6:**
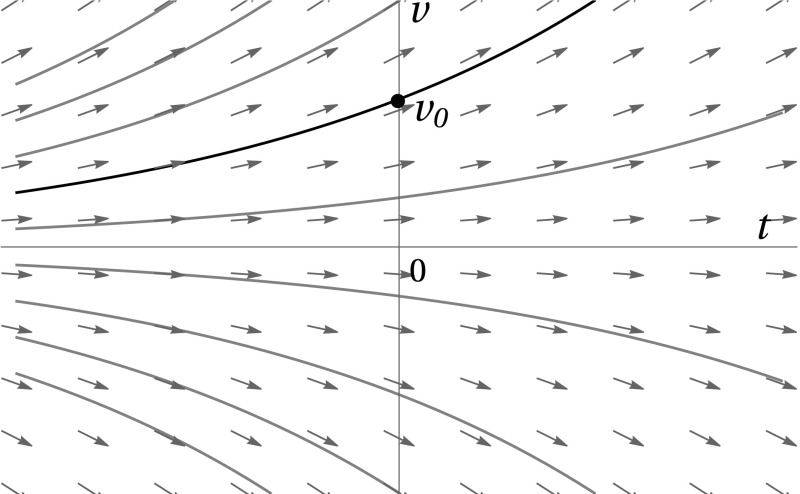
The (*t*, *v*)-coordinate plane, on which the PDE systems (2.12) and (2.13) are solved. The characteristics are integral curves of the vector field (1, *v*), indicated in blue. The black characteristic curve intersects the *v*-axis at $v=v_0$ (colour figure online)

The characteristics of the differential operator $\partial _t + v \partial _v(=\partial _s)$ in (2.19) and (2.32) are the integral curves of the vector field (1, *v*). Geometrically speaking, these characteristic curves foliate the (*t*, *v*)-plane over the *v*-axis. Therefore, each characteristic can be identified by its base point, which is the point where the characteristic curve intersects the *v*-axis, at $v=v_0$; see Eq. (2.17) and Fig. 6.

Equivalently, each characteristic can be represented as a graph over the *t*-axis. Indeed, the differential equation for the *v*-component of a characteristic curve (2.16) can be solved to obtain the natural parametric description of a characteristic ‘fibre’ with base point $v=v_0$, which is given by $(s,v_0 \mathrm{e}^{s})$. Here, the parameter *s* along the characteristic is chosen such that its point of intersection with the *v*-axis lies at $s=0$. Given that choice, it is natural to identify the parameter along the characteristic (*s*) with the time variable (*t*). Hence, the initial conditions in (2.43), which determine a relation between $F^{(0)}_m$ and $F^{(1)}_m$ on the *v*-axis, can be interpreted on every characteristic as

2.44a $$\begin{aligned} \left[ F^{(0)}_0 + F^{(1)}_0\right] _{s=0}&= (v_0+1)^{n_0} \quad \text {and} \end{aligned}$$

2.44b $$\begin{aligned} \left[ F^{(0)}_m + F^{(1)}_m\right] _{s=0}&= 0 \quad \text {for all } m\ge 1, \end{aligned}$$

which again holds for both (2.19) and (2.32).

#### Remark 2.3

For CME systems such as (2.3) and (2.5), it is customary to impose a ‘normalisation’ condition of the form

2.45 $$\begin{aligned} \sum _{m=0}^\infty P^{(0)}_m(t) + P^{(1)}_m(t) = 1, \end{aligned}$$

as $P^{(0)}_m(t)$ and $P^{(1)}_m(t)$ represent probabilities. Recast in the framework of generating functions, recall (2.6), the above normalisation condition yields the boundary condition

2.46 $$\begin{aligned} \left[ F^{(0)}(z,t) + F^{(1)}(z,t)\right] _{z=1} = \left[ F^{(0)}(v,t) + F^{(1)}(v,t)\right] _{v=0} = 1. \end{aligned}$$

It is worthwhile to note that (2.46) is automatically satisfied whenever (2.43) is imposed: by adding the two equations in system (2.12)—or, equivalently, in (2.13)—one sees that $F^{(0)}_m+F^{(1)}_m$ satisfies

2.47 $$\begin{aligned} \partial _s F ^{(0)}_m + \partial _s F^{(1)}_m = \lambda v F^{(1)}_m. \end{aligned}$$

The line $\{v=0\}$ is represented by the ‘trivial’ characteristic, which is identified by $v_0=0$; therefore, on that characteristic, we impose (2.43) to find

2.48a $$\begin{aligned} \partial _s F^{(0)}_m + \partial _s F^{(1)}_m&= 0, \end{aligned}$$

2.48b $$\begin{aligned} \left[ F^{(0)}_0 + F^{(1)}_0\right] _{s=0}&= 1,\quad \text {and} \end{aligned}$$

2.48c $$\begin{aligned} \left[ F^{(0)}_m + F^{(1)}_m\right] _{s=0}&= 0 \quad \text {for all } m\ge 1, \end{aligned}$$

which implies that $F^{(0)}_0 + F^{(1)}_0 = 1$ for all *s*, as well as that $F^{(0)}_m + F^{(1)}_m$ vanishes identically for all $m \ge 1$. Substituting these results into the power series representation for $F^{(j)}(z,t)$ ($j=0,1$) in (2.11), we obtain (2.46).

At this point, it is important to observe that Eq. (2.43) determines a *line* of initial conditions in the phase spaces of the dynamical systems (2.19) and (2.32). Therefore, at every order in $\delta$, we can only fix *one* of the two free parameters that arise in the solution of the corresponding differential equations. In particular, (2.43) only fixes either $c_1$ or $c_2$ in (2.26), and either $c_3$ or $c_4$ in (2.29), and so forth. That indeterminacy motivates us to introduce a new parameter $\chi$, which is defined as follows:

#### Definition 2.4

Consider the CME systems (2.3) and (2.5). We define $\chi (n_0)$ to be the probability that the gene modelled by the reaction scheme in (2.1) is switched *off* at time $t=0$, given an initial protein number $n_0$.

Definition 2.4 immediately specifies initial conditions for systems (2.3) and (2.5) via

2.49 $$\begin{aligned} P^{(0)}_n(0) = \chi (n_0)\,\delta _{n,n_0}\qquad \text {and}\qquad P^{(1)}_n(0) = (1-\chi (n_0))\,\delta _{n,n_0}; \end{aligned}$$

the above expression, in turn, provides us with a complete set of initial conditions for the PDE systems (2.7) and (2.8), to wit

2.50a $$\begin{aligned} F^{(0)}(v,0)&= \chi (n_0)\,(v+1)^{n_0} \quad \text {and} \end{aligned}$$

2.50b $$\begin{aligned} F^{(1)}(v,0)&= (1-\chi (n_0))\,(v+1)^{n_0}. \end{aligned}$$

Here, we allow for the fact that $\chi (n_0)$ may depend on other model parameters, and in particular on the autoregulation rates *a* and *r*; for a discussion of alternative options, see Sect. 4.1. We therefore expand $\chi (n_0)$ as a power series in $\delta$,

2.51 $$\begin{aligned} \chi (n_0) = \sum _{m=0}^\infty \delta ^m \chi _m(n_0). \end{aligned}$$

The above expansion can be used to infer a complete set of initial conditions for the PDE systems (2.12) and (2.13), yielding

2.52a $$\begin{aligned} F^{(0)}_m(v,0)&= \chi _m\,(v+1)^{n_0} \quad \text {for all }m \ge 0, \end{aligned}$$

2.52b $$\begin{aligned} F^{(1)}_0(v,0)&= (1-\chi _0)\,(v+1)^{n_0}, \quad \text {and} \end{aligned}$$

2.52c $$\begin{aligned} F^{(1)}_m(v,0)&= -\chi _m\,(v+1)^{n_0} \quad \text {for all } m\ge 1. \end{aligned}$$

By the same reasoning that inferred (2.44) from (2.43), we can conclude that the complete set of initial conditions for the dynamical systems (2.19) and (2.32) is given by

2.53a $$\begin{aligned} \left[ F^{(0)}_m\right] _{s=0}&= \chi _m\,(v_0+1)^{n_0} \quad \text {for all }m \ge 0, \end{aligned}$$

2.53b $$\begin{aligned} \left[ F^{(1)}_0\right] _{s=0}&= (1-\chi _0)\,(v_0+1)^{n_0}, \quad \text {and} \end{aligned}$$

2.53c $$\begin{aligned} \left[ F^{(1)}_m\right] _{s=0}&= -\chi _m\,(v_0+1)^{n_0} \quad \text {for all } m\ge 1. \end{aligned}$$

We can now use the conditions in (2.53) to determine the free constants $c_{1}$ and $c_{2}$ in (2.26), which yields

2.54a $$\begin{aligned} c_1&= (1+v_0)^{n_0} \mathrm{e}^{-\lambda v_0}\frac{\kappa _b}{\kappa _f+\kappa _b} \Bigg ( {}_1 F_1(-\kappa _b,1-\kappa _f-\kappa _b,\lambda v_0) \nonumber \\&\quad + \frac{\chi _0 \lambda v_0}{1-\kappa _f-\kappa _b} {}_1 F_1(1-\kappa _b,2-\kappa _f-\kappa _b,\lambda v_0)\Bigg )\quad \text {and} \end{aligned}$$

2.54b $$\begin{aligned} c_2&= (1+v_0)^{n_0} \mathrm{e}^{-\lambda v_0}\frac{(\lambda v_0)^{\kappa _f+\kappa _b}}{\kappa _f+\kappa _b}\Bigg \{[\chi _0(\kappa _f+\kappa _b)-\kappa _b] {}_1 F_1(\kappa _f,1+\kappa _f+\kappa _b,\lambda v_0) \nonumber \\&\quad + \frac{\kappa _f \chi _0 \lambda v_0}{1+\kappa _f+\kappa _b} {}_1 F_1(1+\kappa _f,2+\kappa _f+\kappa _b,\lambda v_0)\Bigg \}. \end{aligned}$$

Analogously, for $\hat{c}_{1}$ and $\hat{c}_{2}$ in (2.35), we obtain

2.55 $$\begin{aligned} \hat{c}_1 = c_1 \frac{\kappa _f}{\kappa _b}\qquad \text {and}\qquad \hat{c}_2 = c_2, \end{aligned}$$

with $c_{1}$ and $c_{2}$ as in (2.54). Using conversion formulas found in §13.3(i) of NIST Digital Library of Mathematical Functions , one can show that the expressions resulting from (2.35) and (2.36) match those in (2.27) and (2.26), respectively, as expected; see also Eq. (A.1). Explicit expressions for the integration limits $c_{3}$ and $c_{4}$ in (2.29), as well as for the corresponding limits $\hat{c}_{3}$ and $\hat{c}_{4}$ in (2.38), can be found in Appendix A.

### Inverse transformation

The final step towards providing explicit solutions to the PDE systems (2.7) and (2.8) consists in interpreting the solutions to the dynamical systems (2.19) and (2.32), with initial conditions as in (2.53), as solutions to the PDE systems (2.12) and (2.13), respectively. To that end, we again consider the corresponding characteristics from a geometric viewpoint.

As mentioned in Sect. 2.4, the (*t*, *v*)-coordinate plane is foliated by characteristics, which are the integral curves of the vector field (1, *v*); recall Fig. 6. Hence, any point (*t*, *v*) lies on a unique characteristic; flowing backward along that characteristic to its intersection with the *v*-axis, we can determine the corresponding base point $v_0$ by the inverse transformation

2.56 $$\begin{aligned} (t,v) \mapsto v_0(t,v) = v\mathrm{e}^{-t}, \end{aligned}$$

since we have identified the parameter along the characteristic (*s*) with the time variable (*t*).

To determine the value of $F^{(j)}_m(v,t)$ ($j=0,1$), interpreted as a solution to the PDE system (2.12) or (2.13), we proceed as follows. We first apply the inverse transformation in (2.56) to establish on which characteristic the coordinate pair (*t*, *v*) lies. For that characteristic, identified by its base point $v_0$, we then find the solution to the dynamical system (2.19) or (2.32), which is a function of *s* and $v_0$. Next, we substitute $s = t$ and $v_0 = v\mathrm{e}^{-t}$ into that solution to obtain an explicit expression for the solution to the PDE system (2.12) or (2.13):

2.57 $$\begin{aligned} F^{(j)}_m(v,t)\;\text {[as solution to } (2.12) \text { or } (2.13)\text {]} = \left[ F^{(j)}_m\right] _{(s,v_0)=(t,v \mathrm{e}^{-t})}, \end{aligned}$$

where $F^{(j)}_m$ ($j=0,1$) on the right-hand side denotes the solution to (2.19) or (2.32), with initial conditions as in (2.53). Lastly, we substitute $F^{(j)}_m(v,t)$ into the power series in (2.11) to obtain an explicit solution to (2.7) or (2.8), to satisfactory order in $\delta$.

#### Remark 2.5

The geometric interpretation of characteristics that was used to motivate the inverse transformation in (2.56) also shows that the introduction of the new system parameter $\chi$ in Definition 2.4 is *necessary* for the generating functions $F^{(j)}$ to be determined uniquely as solutions to (2.7) or (2.8), even if we are only interested in their sum $F^{(0)}(v,t) + F^{(1)}(v,t)$. The crucial point is that any free constants obtained in the process of solving the dynamical systems (2.19) and (2.32)—or, equivalently, their second-order one-dimensional counterparts (2.20) and (2.33), respectively—are constant in *s*. In other words, they are constant along the particular characteristic on which the dynamical system is solved. These constants—such as e.g. $c_{1}$ and $c_{2}$ in (2.26)—can, and generally will, depend on the base point $v_0$ of the characteristic; see for example (2.54). The inverse transformation in (2.56) that is used to reconstruct the solution to the original PDE from that of the corresponding dynamical system would then yield undetermined functions $c(v\,\mathrm{e}^{-t})$ in the resulting solutions to (2.7) and (2.8), respectively.

### Summary of main result

To summarise Sect. 2, we combine the analysis of the previous subsections to state our main result.

*Main result:* The PDE system (2.7) can be solved for sufficiently small autoactivation rates *a*; see Assumption 2.2. Its solutions $F^{(j)}(z,t)$ ($j=0,1$) are expressed as power series in the small parameter $\delta$; recall (2.11). The coefficients $F^{(j)}_m(z,t)$ in these series, written in terms of the shifted variable *v* defined in (2.15), can be found by

1. solving recursively the second-order ODE (2.20) and using the identity in (2.24), incorporating the initial conditions in (2.53);
2. and, subsequently, applying the inverse transformation in (2.56) to the resulting solutions.

Likewise, we can solve the PDE system (2.8) for sufficiently small autorepression rates *r*; see Assumption 2.2. Its solutions $F^{(j)}(z,t)$ are again expressed as power series in the small parameter $\delta$; cf. (2.11). The coefficients $F^{(j)}_m(z,t)$ in these series, written in terms of the shifted variable *v* defined in (2.15), can be found by

1. solving recursively the second-order ODE (2.33) and using the identity in (2.34), incorporating the initial conditions in (2.53);
2. and, subsequently, applying the inverse transformation in (2.56) to the resulting solutions.

To illustrate the procedure described above, we state the resulting explicit expressions for the leading-order solution to (2.7)—or, equivalently, to (2.8)—in terms of the original variables *z* and *t*:

2.58a $$\begin{aligned} F^{(0)}_0(z,t)&= \big [1+(z-1)\mathrm{e}^{-t}\big ]^{n_0} \mathrm{e}^{-\lambda (z-1)\mathrm{e}^{-t}} \frac{\kappa _b}{\kappa _f+\kappa _b}\nonumber \\&\quad \Bigg [{}_1 F_1\big (-\kappa _b,1-\kappa _f-\kappa _b,\lambda (z-1)\mathrm{e}^{-t}\big ) \nonumber \\&\quad +\frac{\chi _0 \lambda (z-1)\mathrm{e}^{-t}}{1-\kappa _f-\kappa _b} {}_1 F_1\big (1-\kappa _b,2-\kappa _f-\kappa _b,\lambda (z-1)\mathrm{e}^{-t}\big )\Bigg ] \nonumber \\&\quad \times {}_1 F_1(\kappa _f,1+\kappa _f+\kappa _b,\lambda (z-1)) \nonumber \\&\quad -\big [1+(z-1)\mathrm{e}^{-t}\big ]^{n_0} \mathrm{e}^{-\lambda (z-1)\mathrm{e}^{-t}}\frac{\mathrm{e}^{-(\kappa _f+\kappa _b)t}}{\kappa _f+\kappa _b}\nonumber \\&\quad \times \Bigg \{[\chi _0(\kappa _f+\kappa _b)-\kappa _b] {}_1 F_1\big (\kappa _f,1+\kappa _f+\kappa _b,\lambda (z-1)\mathrm{e}^{-t}\big ) \nonumber \\&\quad +\frac{\kappa _f \chi _0 \lambda (z-1)\mathrm{e}^{-t}}{1+\kappa _f+\kappa _b} {}_1 F_1\big (1+\kappa _f,2+\kappa _f+\kappa _b,\lambda (z-1)\mathrm{e}^{-t}\big )\Bigg \} \nonumber \\&\quad \times {}_1 F_1(-\kappa _b,1-\kappa _f-\kappa _b,\lambda (z-1))\quad \text {and} \end{aligned}$$

2.58b $$\begin{aligned} F^{(1)}_0(z,t)&=\big [1+(z-1)\mathrm{e}^{-t}\big ]^{n_0} \mathrm{e}^{-\lambda (z-1)\mathrm{e}^{-t}} \frac{\kappa _f}{\kappa _f+\kappa _b}\nonumber \\&\quad \times \Bigg [ {}_1 F_1\big (-\kappa _b,1-\kappa _f-\kappa _b,\lambda (z-1)\mathrm{e}^{-t}\big ) \nonumber \\&\quad +\frac{\chi _0 \lambda (z-1)\mathrm{e}^{-t}}{1-\kappa _f-\kappa _b} {}_1 F_1\big (1-\kappa _b,2-\kappa _f-\kappa _b,\lambda (z-1)\mathrm{e}^{-t}\big )\Bigg ] \nonumber \\&\quad \times {}_1 F_1(1+\kappa _f,1+\kappa _f+\kappa _b,\lambda (z-1)) \nonumber \\&\quad +\big [1+(z-1)\mathrm{e}^{-t}\big ]^{n_0} \mathrm{e}^{-\lambda (z-1)\mathrm{e}^{-t}}\frac{\mathrm{e}^{-(\kappa _f+\kappa _b)t}}{\kappa _f+\kappa _b}\nonumber \\&\quad \times \Bigg \{[\chi _0(\kappa _f+\kappa _b)-\kappa _b] {}_1 F_1\big (\kappa _f,1+\kappa _f+\kappa _b,\lambda (z-1)\mathrm{e}^{-t}\big ) \nonumber \\&\quad +\frac{\kappa _f \chi _0 \lambda (z-1)\mathrm{e}^{-t}}{1+\kappa _f+\kappa _b} {}_1 F_1\big (1+\kappa _f,2+\kappa _f+\kappa _b,\lambda (z-1)\mathrm{e}^{-t}\big )\Bigg \}\nonumber \\&\quad \times {}_1 F_1(1-\kappa _b,1-\kappa _f-\kappa _b,\lambda (z-1)). \end{aligned}$$

Note that similar expressions were derived by Iyer-Biswas et al. (2009), where an analogous generating function approach was applied under the assumption that the gene is initially inactive—i.e. that $\chi (n_0)=1$, see Definition 2.4—as well as that the initial protein number $n_0$ is zero. With these choices, the expression for $F^{(0)}_0+F^{(1)}_0$ from (2.58) can be seen to coincide with that found in Equations (5) through (7) of Iyer-Biswas et al. (2009), using §13.3.4 and §13.2.39 of NIST Digital Library of Mathematical Functions .

## Model B: gene expression with explicit transcription

In this section, we apply our analytical method to model B, a stochastic gene expression model presented by Shahrezaei and Swain (2008a) which explicitly incorporates the transcription stage in the expression process, as well as DNA switching; see also Fig. 4. In the original article by Shahrezaei and Swain (2008a), a generating function approach was used to obtain analytical expressions for the time-independent (‘stationary’) solution to the model. For a visual guide to the upcoming analysis, the reader is again referred to Fig. 5.

### Stochastic model and CME

The model for stochastic gene expression considered here is given by the reaction scheme3.1The modelled gene can hence switch between inactive and active states which are denoted by *D* and $D^*$, respectively, with corresponding switching rates $k_0$ and $k_1$. The active gene is transcribed to mRNA (*M*) with rate $\nu _0$; mRNA is translated to protein (*P*) with rate $\nu _1$. Finally, mRNA decays with rate $d_0$, while protein decays with rate $d_1$.

As in model A, autoregulatory terms can be added to the core reaction scheme in (3.1). Since both mRNA and protein are modelled explicitly, we can identify four distinct autoregulatory mechanisms, in analogy to those in (2.2a) and (2.2b):

3.2a $$\begin{aligned} D + M&{\mathop {\rightarrow }\limits ^{a_M}} D^* + M&\text {(autoactivation through mRNA)}, \end{aligned}$$

3.2b $$\begin{aligned} D^* + M&{\mathop {\rightarrow }\limits ^{r_M}} D + M&\text {(autorepression through mRNA)}, \end{aligned}$$

3.2c $$\begin{aligned} D + P&{\mathop {\rightarrow }\limits ^{a_P}} D^* + P&\text {(autoactivation through protein)}, \end{aligned}$$

3.2d $$\begin{aligned} D^* + P&{\mathop {\rightarrow }\limits ^{r_P}} D + P&\text {(autorepression through protein)}. \end{aligned}$$

Autoactivation can be achieved either by mRNA or by protein, with rates $a_M$ or $a_P$, respectively; similarly, autorepression can occur either through mRNA or through protein, with respective rates $r_M$ or $r_P$.

The CME system associated to the reaction scheme in (3.1) is given by

3.3a $$\begin{aligned} \frac{\text {d} P^{(0)}_{m,n}}{\text {d} t}&= -\kappa _0 P^{(0)}_{m,n} + \kappa _1 P^{(1)}_{m,n} + \gamma \left[ (m+1)P^{(0)}_{m+1,n}-m P^{(0)}_{m,n}\right] \nonumber \\&\quad + \left[ (n+1)P^{(0)}_{m,n+1}- n P^{(0)}_{m,n}\right] \nonumber \\&\quad + \gamma \mu \left( m P^{(0)}_{m,n-1} - m P^{(0)}_{m,n}\right) , \end{aligned}$$

3.3b $$\begin{aligned} \frac{\text {d} P^{(1)}_{m,n}}{\text {d} t}&= \kappa _0 P^{(0)}_{m,n} - \kappa _1 P^{(1)}_{m,n} + \gamma \left[ (m+1)P^{(1)}_{m+1,n}-m P^{(1)}_{m,n}\right] \nonumber \\&\quad + \left[ (n+1)P^{(1)}_{m,n+1}- n P^{(1)}_{m,n}\right] \nonumber \\&\quad + \gamma \mu \left( m P^{(1)}_{m,n-1} - m P^{(1)}_{m,n}\right) + \lambda \left( P^{(1)}_{m-1,n}-P^{(1)}_{m,n}\right) . \end{aligned}$$

Here, $P^{(j)}_{m,n}(t)$ ($j=0,1$) represents the probability of *m* mRNA and *n* protein being present at time *t* while the gene is either inactive (0) or active (1). As in (2.3) and (2.5), the time variable is nondimensionalised by the protein decay rate $d_1$; other model parameters are scaled as

3.4 $$\begin{aligned} \kappa _0 = \frac{k_0}{d_1},\quad \kappa _1 = \frac{k_1}{d_1},\quad \gamma = \frac{d_0}{d_1},\quad \lambda = \frac{\nu _0}{d_1},\quad \text {and}\quad \mu = \frac{\nu _1}{d_0}. \end{aligned}$$

We note that the above scaling was also used by Shahrezaei and Swain (2008a). The effects of incorporating the autoregulatory mechanisms in (3.2) into the CME system (3.3) are specified in Table 1.

**Table 1:** Contribution to the right-hand sides of (3.3) that is due to incorporation of the autoregulatory mechanisms in (3.2)

Autoregulation type	Contribution to (3.3a)	Contribution to (3.3b)
mRNA autoactivation	$-\,\frac{a_M}{d_1} m P^{(0)}_{m,n}$	$+\, \frac{a_M}{d_1} m P^{(0)}_{m,n}$
mRNA autorepression	$+\,\frac{r_M}{d_1} m P^{(1)}_{m,n}$	$-\, \frac{r_M}{d_1} m P^{(1)}_{m,n}$
Protein autoactivation	$-\,\frac{a_P}{d_1} n P^{(0)}_{m,n}$	$+\,\frac{a_P}{d_1} n P^{(0)}_{m,n}$
Protein autorepression	$+\,\frac{r_P}{d_1} n P^{(1)}_{m,n}$	$-\,\frac{r_P}{d_1} n P^{(1)}_{m,n}$

### Generating function PDE

Since the probabilities $P^{(j)}_{m,n}(t)$ ($j=0,1$) in (3.3) depend on both the mRNA number *m* and the protein number *n*, we introduce probability-generating functions that are defined by double asymptotic series:

3.5 $$\begin{aligned} F^{(j)}(w,z,t) = \sum _{m=0}^\infty \sum _{n=0}^\infty w^m z^n P^{(j)}_{m,n}(t)\quad \text {for }j=0,1. \end{aligned}$$

For coefficients $P^{(j)}_{m,n}(t)$ that obey the CME system (3.3), the associated generating functions $F^{(j)}(w,z,t)$ satisfy

3.6a $$\begin{aligned}&\partial _t F^{(0)} + (z-1) \partial _z F^{(0)} + \gamma (w-1)\partial _w F^{(0)} - \gamma \mu (z-1) w \partial _w F^{(0)}\nonumber \\&\quad = - \kappa _0 F^{(0)} + \kappa _1 F^{(1)}, \end{aligned}$$

3.6b $$\begin{aligned}&\partial _t F^{(1)} + (z-1) \partial _z F^{(1)} + \gamma (w-1)\partial _w F^{(1)} - \gamma \mu (z-1) w \partial _w F^{(1)}\nonumber \\&\quad = \kappa _0 F^{(0)} - \kappa _1 F^{(1)}+ \lambda (w-1) F^{(1)}. \end{aligned}$$

The effects of incorporating the autoregulatory mechanisms in (3.2) into system (3.6) are specified in Table 2.

**Table 2:** Contribution to the right-hand sides of (3.6) that is due to incorporation of the autoregulatory mechanisms in (3.2)

Autoregulation type	Contribution to (3.6a)	Contribution to (3.6b)
mRNA autoactivation	$-\,\frac{a_M}{d_1} w\partial _w F^{(0)}$	$+\, \frac{a_M}{d_1} w\partial _w F^{(0)}$
mRNA autorepression	$+\,\frac{r_M}{d_1} w\partial _w F^{(1)}$	$-\, \frac{r_M}{d_1} w\partial _w F^{(1)}$
Protein autoactivation	$-\,\frac{a_P}{d_1} z\partial _z F^{(0)}$	$+\, \frac{a_P}{d_1} z\partial _z F^{(0)}$
Protein autorepression	$+\,\frac{r_P}{d_1} z\partial _z F^{(1)}$	$-\, \frac{r_P}{d_1} z\partial _z F^{(1)}$

### Dynamical systems analysis

As before, the PDE system (3.6) for the generating function can be reformulated as a system of ODEs via the method of characteristics. The differential operator $\partial _t + (z-1)\partial _z +\gamma (w-1)\partial _w-\gamma \mu (z-1)w \partial _w$ now gives rise to the characteristic system

3.7a $$\begin{aligned} \dot{u}&= \gamma [u - \mu v(u+1)], \end{aligned}$$

3.7b $$\begin{aligned} \dot{v}&= v, \end{aligned}$$

where $\dot{u} = \frac{\text {d} u}{\text {d} s}$ is the derivative along the characteristic, which is parametrised by *s*. For simplicity, we have introduced the new variables *u* and *v*, which are defined as

3.8 $$\begin{aligned} u = w-1\qquad \text {and}\qquad v = z-1, \end{aligned}$$

respectively. On the resulting characteristics, Eq. (3.6) yields the system of ODEs

3.9a $$\begin{aligned} \dot{F}^{(0)}&= -\kappa _0 F^{(0)} + \kappa _1 F^{(1)}, \end{aligned}$$

3.9b $$\begin{aligned} \dot{F}^{(1)}&= \kappa _0 F^{(0)} - \kappa _1 F^{(1)} +\lambda u F^{(1)}. \end{aligned}$$

First, we note that the characteristic system (3.7) can be solved explicitly in terms of the incomplete Gamma function $\Gamma (a,z)$, see §8.2(i) of NIST Digital Library of Mathematical Functions ), yielding

3.10a $$\begin{aligned} u(s)&= \mathrm{e}^{\gamma (s-\mu v_0 \mathrm{e}^{s})}\Big \{u_0 \mathrm{e}^{\gamma \mu v_0} + \left( -\gamma \mu v_0\right) ^\gamma \nonumber \\&\qquad \big [\Gamma (1-\gamma ,-\gamma \mu v_0) - \Gamma (1-\gamma ,-\gamma \mu v_0 \mathrm{e}^s)\big ]\Big \}, \end{aligned}$$

3.10b $$\begin{aligned} v(s)&= v_0 \mathrm{e}^s. \end{aligned}$$

However, due to its complex nature, the expression for *u*(*s*) given in (3.10a) cannot be used to obtain explicit expressions for the generating functions $F^{(j)}$ that solve system (3.9). Therefore, inspired by the analysis by Shahrezaei and Swain (2008a) and Popović et al. (2016), we make the following assumption:

#### Assumption 3.1

We assume that the decay rate of protein ($d_1$) is *smaller* than the decay rate of mRNA ($d_0$); specifically, we write

3.11 $$\begin{aligned} \frac{d_0}{d_1} = \gamma = \frac{1}{\varepsilon }, \end{aligned}$$

where $0<\varepsilon <1$ is sufficiently small.

The resulting scale separation between mRNA and protein decay rates is well-documented in many microbial organisms, including in bacteria and yeast (Shahrezaei and Swain 2008a; Yu et al. 2006).

For clarity of presentation, we make an additional assumption here.

#### Assumption 3.2

We assume that all other model parameters $\kappa _0$, $\kappa _1$, $\lambda$, and $\mu$, as defined in (3.4), are $\mathcal {O}(1)$ in $\varepsilon$.

#### Remark 3.3

Although Assumption 3.2 is not strictly necessary for the upcoming analysis, it is beneficial. It is worthwhile to note that the analytical scheme presented in this section can be applied in a straightforward fashion in cases where Assumption 3.2 fails, which is particularly relevant in relation to previous work (Shahrezaei and Swain 2008a; Feigelman et al. 2015), where the CME system (3.3) is studied for parameter values far beyond the range implied by Assumption 3.2.

Using Assumption 3.1, we can write the characteristic system (3.7) as

3.12a $$\begin{aligned} \varepsilon \dot{u}&= u - \mu v(u+1), \end{aligned}$$

3.12b $$\begin{aligned} \dot{v}&= v. \end{aligned}$$

Since $\varepsilon$ is assumed to be small, we can classify (3.12) as a *singularly* perturbed *slow-fast* system in standard form; see Kuehn (2015). A comprehensive slow-fast analysis of Eq. (3.12) was carried out by Popović et al. (2016); we highlight some relevant aspects of that analysis here.

**Fig. 7:**
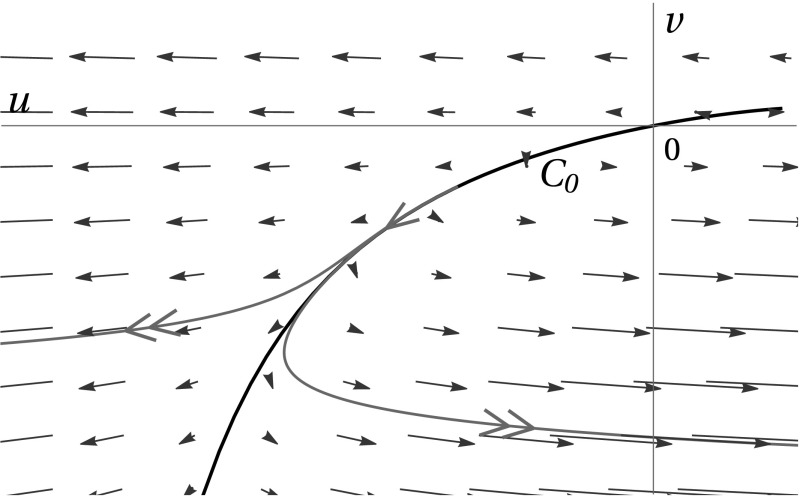
Phase space dynamics of systems (3.12) and (3.13). The slow flow along $C_0$ is indicated by single arrows; the fast dynamics transverse to $C_0$ are denoted by double arrows

System (3.12) gives rise to a critical manifold $C_0 = \left\{ (u,v)\, \big |\, u = \frac{\mu v}{1-\mu v}\right\}$. If $\varepsilon$ is asymptotically small, orbits of (3.12) can be separated into slow and fast segments, using Fenichel’s geometric singular perturbation theory (Kuehn 2015). The critical manifold $C_0$ is normally repelling; in other words, orbits converge to $C_0$ in backward time at an exponential rate. For initial conditions asymptotically close to $C_0$, orbits initially follow $C_0$ closely for some time, after which they move away from $C_0$ under the fast dynamics; see also Fig. 7.

To analyse the fast dynamics of system (3.12), we introduce the fast variable $\sigma = \frac{s}{\varepsilon }$; in terms of $\sigma$, (3.12) is hence expressed as

3.13a $$\begin{aligned} u'&= u - \mu v(u+1), \end{aligned}$$

3.13b $$\begin{aligned} v'&= \varepsilon v, \end{aligned}$$

where $u' = \frac{\text {d} u}{\text {d} \sigma }$. We can solve (3.13b) explicitly and write the result as a power series in $\varepsilon$, which yields

3.14 $$\begin{aligned} v(\sigma ) = v_0 \mathrm{e}^{\varepsilon \sigma } = v_0 \sum _{n=0}^\infty \varepsilon ^n \frac{\sigma ^n}{n!}. \end{aligned}$$

Expressing the solution to (3.13a) as a power series in $\varepsilon$, as well, i.e. writing

3.15 $$\begin{aligned} u(\sigma ) = \sum _{n=0}^\infty \varepsilon ^n \hat{u}_n(\sigma ), \end{aligned}$$

substituting (3.15) into (3.13a), and making use of (3.14), we find

3.16 $$\begin{aligned} \frac{\text {d} \hat{u}_n}{\text {d} \sigma } = \hat{u}_n - \mu v_0 \frac{\sigma ^n}{n!} \left[ 1+ \sum _{l=0}^n \frac{\sigma ^{-l} n!}{(n-l)!} \hat{u}_l \right] , \end{aligned}$$

with initial conditions

3.17 $$\begin{aligned} \hat{u}_0(0) = u_0\qquad \text {and}\qquad \hat{u}_n(0) = 0 \quad \text {for }n\ge 1. \end{aligned}$$

The first two terms in (3.15) are thus given by

3.18a $$\begin{aligned} \hat{u}_0(\sigma )&= \frac{\mu v_0}{1-\mu v_0} +\left( u_0 -\frac{\mu v_0}{1-\mu v_0}\right) \mathrm{e}^{(1-\mu v_0)\sigma }\quad \text {and} \end{aligned}$$

3.18b $$\begin{aligned} \hat{u}_1(\sigma )&= \frac{\mu v_0}{(1-\mu v_0)^2}\left[ \frac{1-\mathrm{e}^{(1-\mu v_0)\sigma }}{1-\mu v_0}+\sigma \right] - \left( u_0 - \frac{\mu v_0}{1-\mu v_0}\right) \mu v_0\frac{\sigma ^2}{2}\mathrm{e}^{(1-\mu v_0)\sigma }. \end{aligned}$$

We now employ the expansion in (3.15) to obtain explicit expressions for the generating functions $F^{(j)}$ ($j=0,1$). In the fast variable $\sigma = \frac{s}{\varepsilon }$, system (3.9) takes the form

3.19a $$\begin{aligned} F^{(0)\prime }&= -\varepsilon \kappa _0 F^{(0)} + \varepsilon \kappa _1 F^{(1)}, \end{aligned}$$

3.19b $$\begin{aligned} F^{(1)\prime }&= \varepsilon \kappa _0 F^{(0)} - \varepsilon \kappa _1 F^{(1)} +\varepsilon \lambda u F^{(1)}. \end{aligned}$$

As in the analysis of model A, recall Sect. 2.3, we rewrite system (3.19) as a second-order ODE for $F^{(0)}$ to find

3.20 $$\begin{aligned} \frac{\text {d}^2}{\text {d} \sigma ^2} F^{(0)} + \varepsilon (\kappa _0+\kappa _1 - \lambda u) \frac{\text {d}}{\text {d} \sigma } F^{(0)} - \varepsilon ^2 \lambda \kappa _0 u F^{(0)} = 0. \end{aligned}$$

Next, we use (3.19a) to express $F^{(1)}$ in terms of $F^{(0)}$ as

3.21 $$\begin{aligned} F^{(1)} = \frac{1}{\varepsilon \kappa _1} \left( F^{(0)\prime } + \varepsilon \kappa _0 F^{(0)}\right) . \end{aligned}$$

To incorporate the expansion for *u* in (3.15), we also expand $F^{(j)}$ ($j=0,1$) in powers of $\varepsilon$, writing

3.22 $$\begin{aligned} F^{(j)}(\sigma ) = \sum _{n=0}^\infty \varepsilon ^nF^{(j)}_n(\sigma ). \end{aligned}$$

Substitution of (3.22) into (3.20) then yields

3.23a $$\begin{aligned} \frac{\text {d}^2}{\text {d} \sigma ^2} F^{(0)}_0&= 0, \end{aligned}$$

3.23b $$\begin{aligned} \frac{\text {d}^2}{\text {d} \sigma ^2} F^{(0)}_1 + (\kappa _0+\kappa _1 - \lambda \hat{u}_0) \frac{\text {d}}{\text {d} \sigma } F^{(0)}_0&= 0, \end{aligned}$$

and

3.24 $$\begin{aligned} \frac{\text {d}^2}{\text {d} \sigma ^2} F^{(0)}_n + (\kappa _0+\kappa _1 - \lambda \hat{u}_0) \frac{\text {d}}{\text {d} \sigma } F^{(0)}_{n+1} -\lambda \sum _{k=0}^n \left( \hat{u}_{n+1-k} \frac{\text {d}}{\text {d} \sigma } + \kappa _0\, \hat{u}_{n-k}\right) F^{(0)}_k = 0 \end{aligned}$$

for $n \ge 2$. By combining (3.21) with (3.22), we obtain

3.25 $$\begin{aligned} \frac{\text {d}}{\text {d} \sigma } F^{(0)}_0 = 0 \end{aligned}$$

and

3.26 $$\begin{aligned} F^{(1)}_n = \frac{\kappa _0}{\kappa _1} F^{(0)}_n + \frac{1}{\kappa _1} \frac{\text {d}}{\text {d} \sigma } F^{(0)}_{n+1} \end{aligned}$$

for $n \ge 0$. We can solve Eqs. (3.23) and (3.24) iteratively—taking into account the additional condition on $F^{(0)}_0$ in (3.25)—to find

3.27a $$\begin{aligned} F^{(0)}_0&= f_0, \end{aligned}$$

3.27b $$\begin{aligned} F^{(0)}_1&= f_1 + g_1 \sigma ,\quad \text {and} \end{aligned}$$

3.27c $$\begin{aligned} F^{(0)}_2&= f_2 + g_2 \sigma + \frac{\lambda (f_0 \kappa _0 + g_1)}{1-\mu v_0}\left[ \frac{\mathrm{e}^{(1-\mu v_0)\sigma }}{1-\mu v_0}\left( u_0 - \frac{\mu v_0}{1-\mu v_0}\right) + \mu v_0\frac{\sigma ^2}{2}\right] \nonumber \\&\quad -g_1(\kappa _0+\kappa _1) \frac{\sigma ^2}{2} \end{aligned}$$

for the first three terms which, upon substitution into (3.26), yields

3.28a $$\begin{aligned} F^{(1)}_0&= \frac{f_0 \kappa _0 + g_1}{\kappa _1}\quad \text {and} \end{aligned}$$

3.28b $$\begin{aligned} F^{(1)}_1&= \frac{f_1 \kappa _0 + g_2}{\kappa _1} + \frac{\lambda (f_0 \kappa _0 + g_1)}{\kappa _1(1-\mu v_0)}\left[ \mathrm{e}^{(1-\mu v_0)\sigma }\left( u_0 - \frac{\mu v_0}{1-\mu v_0}\right) +\sigma \mu v_0\right] -\sigma g_1; \end{aligned}$$

here, $f_i$ and $g_i$ are free constants, to be determined by initial conditions; see Sect. 3.4.

Contrary to common practice in the study of slow-fast systems such as (3.19), we forego a detailed analysis of the slow system (3.9), continuing our discussion with the determination of appropriate initial conditions; cf. Sect. 2.4. For details on why the slow dynamics is disregarded, the reader is referred to Remark 3.5.

### Initial conditions and inverse transformation

To complete our analytical method, we discuss the determination of initial conditions and the reconstruction of the solution to the original PDE system (3.6), as was done for model A in Sects. 2.4 and 2.5, respectively.

#### Initial conditions

We follow the reasoning of Sect. 2.4, and determine appropriate initial conditions by considering the original CME system (3.3).

At time $t=0$, we prescribe initial mRNA and protein numbers $m_0$ and $n_0$, respectively. As in Sect. 2.4, we again introduce the parameter $\chi$, which is defined as follows; compare Definition 2.4:

##### Definition 3.4

Consider the CME system (3.3). We define $\chi (m_0,n_0)$ to be the probability that the gene modelled by the reaction scheme in (3.1) is switched *off* at time $t=0$, given initial mRNA and protein numbers $m_0$ and $n_0$, respectively.

For the probabilities $P^{(j)}_{m,n}(t)$ ($j=0,1$), Definition 3.4 implies that

3.29a $$\begin{aligned} P^{(0)}_{m,n}(0)&= \chi \,\delta _{m,m_0} \,\delta _{n,n_0}\quad \text {and} \end{aligned}$$

3.29b $$\begin{aligned} P^{(1)}_{m,n}(0)&= (1-\chi )\delta _{m,m_0} \,\delta _{n,n_0}. \end{aligned}$$

Using the definition of the generating functions $F^{(j)}(w,z,t)$ in (3.5), we find

3.30a $$\begin{aligned} F^{(0)}(w,z,0)&= \chi \,w^{m_0} z^{n_0} = \chi (1+u)^{m_0}(1+v)^{n_0}\quad \text {and} \end{aligned}$$

3.30b $$\begin{aligned} F^{(1)}(w,z,0)&= (1-\chi )w^{m_0} z^{n_0} = (1-\chi ) (1+u)^{m_0}(1+v)^{n_0}, \end{aligned}$$

taking into account the change of variables in (3.8).

Next, we can infer e.g. from (3.10) and well-known properties of the incomplete Gamma function—see §8.2 of NIST Digital Library of Mathematical Functions —that solutions to (3.7) exist globally, i.e. for all *s*. In particular, given an arbitrary triple $(s_*,u_*,v_*)$, we can apply the inverse flow of (3.7) to flow backward $(u_*,v_*)$ for time $s_*$. We conclude that the characteristics of the operator $\partial _t +(z-1)\partial _z + \gamma (w-1)\partial _w -\gamma \mu (z-1)w \partial _w = \partial _t + v \partial _v + \gamma u \partial _u - \gamma \mu (u+1)v \partial _u$, which are given by the orbits of system (3.7), foliate the (*t*, *u*, *v*)-coordinate space over the $\left\{ t=0\right\}$-plane when the parameter along the characteristic (*s*) is identified with the time variable (*t*). We can therefore uniquely identify such a characteristic—interpreted as a fibre over the $\left\{ t=0\right\}$-plane—by its base point. Because orbits of (3.7) provide a parametrisation of the underlying characteristics, the coordinates of that base point are given by $(0,u_0,v_0)$, where $(u_0,v_0)$ are the initial values of the corresponding orbit of (3.7). Hence, the conditions in (3.30) yield, on each characteristic,

3.31a $$\begin{aligned} \left[ F^{(0)}\right] _{s=0}&= \chi (1+u_0)^{m_0}(1+v_0)^{n_0}\quad \text {and} \end{aligned}$$

3.31b $$\begin{aligned} \left[ F^{(1)}\right] _{s=0}&= (1-\chi ) (1+u_0)^{m_0}(1+v_0)^{n_0}. \end{aligned}$$

As $s=0$ implies $\sigma = 0$, we can apply the initial conditions in (3.31) to the solutions of the ODE system (3.19) in order to determine the free constants $f_i$ and $g_i$ in (3.27) and (3.28). Combining the power series expansion in (3.22) with (3.31), we obtain

3.32a $$\begin{aligned} \left[ F^{(0)}_0\right] _{\sigma =0}&= \chi (1+u_0)^{m_0}(1+v_0)^{n_0}, \end{aligned}$$

3.32b $$\begin{aligned} \left[ F^{(1)}_0\right] _{\sigma =0}&= (1-\chi ) (1+u_0)^{m_0}(1+v_0)^{n_0},\quad \text {and} \end{aligned}$$

3.32c $$\begin{aligned} \left[ F^{(j)}_n\right] _{\sigma =0}&= 0 \quad \text {for all } n\ge 1,\text { with }j=0,1, \end{aligned}$$

which implies

3.33a $$\begin{aligned} f_0&= \chi (1+u_0)^{m_0}(1+v_0)^{n_0}, \end{aligned}$$

3.33b $$\begin{aligned} f_1&= 0, \end{aligned}$$

3.33c $$\begin{aligned} f_2&= -(1-\chi ) (1+u_0)^{m_0}(1+v_0)^{n_0} \frac{\lambda \kappa _1}{(1-\mu v_0)^2} \left( u_0 - \frac{\mu v_0}{1-\mu v_0}\right) , \end{aligned}$$

3.33d $$\begin{aligned} g_1&= (\kappa _1(1-\chi ) - \kappa _0 \chi )(1+u_0)^{m_0}(1+v_0)^{n_0},\quad \text {and} \end{aligned}$$

3.33e $$\begin{aligned} g_2&= -(1-\chi ) (1+u_0)^{m_0}(1+v_0)^{n_0}\frac{\lambda \kappa _1}{1-\mu v_0} \left( u_0 - \frac{\mu v_0}{1-\mu v_0}\right) \end{aligned}$$

in (3.27) and (3.28).

#### Inverse transformation

Since the (*t*, *u*, *v*)-coordinate space is foliated by the characteristics of the operator $\partial _t +v \partial _v + \gamma u \partial _u - \gamma \mu (u+1)v \partial _u$, any point (*t*, *u*, *v*) lies on a unique characteristic. Flowing backward along that characteristic to its intersection with the $\left\{ t=0\right\}$-plane, we can determine the corresponding base point $(0,u_0,v_0)$ by inverting the relations in (3.10). Since the dynamics of the *v*-coordinate do not depend on *u*, we may use (2.56) to express $v_0$ in terms of *t* and *v* only. Taking the resulting expression as input for inverting (3.10a), we obtain the inverse characteristic transformation

3.34 $$\begin{aligned} (t,u,v) \mapsto \left( u_0(t,u,v), v_0(t,v)\right) , \end{aligned}$$

with

3.35a $$\begin{aligned} u_0(t,u,v)&= \mathrm{e}^{-\gamma (t+ \mu v \mathrm{e}^{-t})}\left\{ u\, \mathrm{e}^{\gamma \mu v} - \left( -\gamma \mu v\right) ^\gamma \big [\Gamma (1-\gamma ,-\gamma \mu v \mathrm{e}^{-t}) \right. \nonumber \\&\quad \left. - \Gamma (1-\gamma ,-\gamma \mu v )\big ]\right\} \quad \text {and} \end{aligned}$$

3.35b $$\begin{aligned} v_0(t,v)&= v \mathrm{e}^{-t}. \end{aligned}$$

Under Assumption 3.1, we can employ the power series expansion in (3.15), in combination with the recursive set of ODEs in (3.16) and initial conditions as in (3.17), as an alternative to (3.35a) to obtain $u_0$ as a function of *u*, *v* (or $v_0$), and $\sigma$. Rewriting the result as a power series in $\varepsilon$, we find

3.36 $$\begin{aligned} u_0(\sigma ,u,v)&=\frac{\mu v}{1-\mu v} + \mathrm{e}^{(\mu v-1) \sigma }\left( u - \frac{\mu v}{1-\mu v}\right) \nonumber \\&\quad - \varepsilon \mu v \left[ \frac{\mathrm{e}^{(\mu v-1)\sigma }-1}{(1-\mu v)^3} + \frac{\sigma }{(1-\mu v)^2} + \frac{\sigma ^2}{2}\mathrm{e}^{(\mu v -1)\sigma }\left( u - \frac{\mu v}{1-\mu v}\right) \right] \nonumber \\&\quad + \mathcal {O}(\varepsilon ^2); \end{aligned}$$

naturally, (3.14) gives rise to

3.37 $$\begin{aligned} v_0(\sigma ,v) = v_0 \sum _{n=0}^\infty \varepsilon ^n \frac{(-\sigma )^n}{n!}. \end{aligned}$$

Since the independent variable in (3.16) is $\sigma$, and since *s* is naturally identified with *t*, we replace $\sigma$ with $\frac{t}{\varepsilon }$ in (3.36) to obtain a perturbative expansion for $u_0(t,u,v)$.

The solution to the PDE system (3.6) can now be found to satisfactory order in $\varepsilon$ by applying the inverse transformation in (3.34) to the solutions given in (3.27) and (3.28), taking into account the values of $f_i$ and $g_i$ in (3.33). In other words,

3.38 $$\begin{aligned} F^{(j)}(u,v,t)\;\text {[as solution to (}3.6\text {)]} = \left[ \sum _{n=0}^\infty \varepsilon ^n\left[ F^{(j)}_n\right] _{(u_0,v_0) = (u_0(\sigma ,u,v),v \mathrm{e}^{-\varepsilon \sigma })}\right] _{\sigma =\frac{t}{\varepsilon }}, \end{aligned}$$

where $F^{(j)}_n$ ($j=0,1$) on the right-hand side of the above expression denotes the solution to Eqs. (3.23a)–(3.26), with initial conditions as in (3.32).

##### Remark 3.5

The absence of any detailed analysis of the characteristic system in its slow formulation, Eq. (3.12), can be argued as follows, by considering the corresponding phase space, as depicted in Fig. 7.

- For arbitrary initial conditions $(u_0,v_0)$, the dominant dynamics are fast, since the critical manifold $C_0$ is normally repelling. In other words, solutions are generally repelled away from $C_0$ under the fast dynamics.
- All orbits that have their initial conditions on the same Fenichel fibre are *exponentially close* (in $\varepsilon$) to each other near the slow manifold $C_\varepsilon$ that is associated to the critical manifold $C_0$. Therefore, flowing backward from (*u*, *v*) to $(u_0,v_0)$—as expressed through the inverse transformation in (3.34) that yields the corresponding PDE solution—may introduce *exponentially large* terms in the transformation, precluding any sensible series expansion.

Thus, although the construction of a composite ‘(initially) slow—(ultimately) fast’ expression of $F^{(j)}$ as a solution to systems (3.12) and (3.13) certainly makes sense from a dynamical systems perspective, the extreme lack of sensitivity of orbits on their initial conditions $(u_0,v_0)$ may prevent such a composite expansion from being useful for obtaining solutions to the original PDE system (3.6).

##### Remark 3.6

In Sect. 3.3, explicit expressions are given for the expansion of $F^{(0)}$ up to $\mathcal {O}(\varepsilon ^2)$ only, cf. (3.27); similarly, $F^{(1)}$ is approximated to $\mathcal {O}(\varepsilon )$ in (3.28), for the sake of brevity. It is worthwhile to note that a lower bound on the order of the expansion is stipulated by the application; recall Sect. 1.1: the sampling time $\Delta t$ can be considered as a *minimum* time interval over which the results of our analysis should be (reasonably) accurate. To that end, we have to compare $\Delta t$ with $\varepsilon = \frac{1}{\gamma }$, the parameter defining the fast time scale on which the above analytical results have been derived. We can then apply the classical theory of Poincaré expansions (Verhulst 2000) to infer that, if for example $\Delta t = \mathcal {O}(1)$—which implies $\Delta t = \mathcal {O}(\varepsilon ^{-1})$ in the fast time variable $\sigma$—the generating functions $F^{(j})$ should at least be expanded up to $\mathcal {O}(\varepsilon ^2)$ for the resulting approximation to be accurate to $\mathcal {O}(\varepsilon )$.

### Autoregulation

The inclusion of any type of autoregulation into system (3.6)—which is equivalent to the addition of model terms from Table 2 to the right-hand sides of the corresponding equations—precludes the direct application of the method of characteristics, as the resulting partial differential operators in Eqs. (3.6a) and (3.6b) do not coincide anymore. To resolve that complication, we follow the approach of Sect. 2, making the following assumption:

#### Assumption 3.7

We assume that the autoregulation rates $a_M$, $r_M$, $a_P$, and $r_P$, as defined in (3.2), are *small* in comparison with the protein decay rate $d_1$; specifically, we write

3.39 $$\begin{aligned} a_M=\alpha _M \,d_1\,\delta ,\quad r_M=\rho _M \,d_1\,\delta ,\quad a_P=\alpha _P \,d_1\,\delta ,\quad \text {and}\quad r_P=\rho _P \,d_1\,\delta , \end{aligned}$$

where $0<\delta <1$ is sufficiently small.

Next, we expand the generating functions $F^{(j)}$ ($j=0,1$) as power series in $\delta$; recall (2.11):

3.40 $$\begin{aligned} F^{(j)}(z,w,t) = \sum _{m=0}^\infty \delta ^m F^{(j)}_m(z,w,t). \end{aligned}$$

To demonstrate the procedure, we include mRNA autoactivation in (3.6), see again Table 2; the analysis of the remaining autoregulatory mechanisms can be performed in a similar fashion. Substitution of (3.40) now yields

3.41a $$\begin{aligned}&\left[ \partial _t + (z-1) \partial _z + \gamma (w-1)\partial _w - \gamma \mu (z-1) w \partial _w \right] F^{(0)}_m \nonumber \\&\quad = - \kappa _0 F^{(0)}_m + \kappa _1 F^{(1)}_m - \alpha _M w \partial _w F^{(0)}_{m-1}, \end{aligned}$$

3.41b $$\begin{aligned}&\left[ \partial _t + (z-1) \partial _z + \gamma (w-1)\partial _w - \gamma \mu (z-1) w \partial _w \right] F^{(1)}_m \nonumber \\&\quad = \kappa _0 F^{(0)}_m - \kappa _1 F^{(1)}_m + \lambda (w-1) F^{(1)}_m + \alpha _M w \partial _w F^{(0)}_{m-1}; \end{aligned}$$

cf. (2.12). System (3.41) is then amenable to the method of characteristics. In fact, employing the same characteristics as in the unperturbed setting, recall (3.7), we find

3.42 $$\begin{aligned} \partial _t + (z-1) \partial _z + \gamma (w-1)\partial _w - \gamma \mu (z-1) w \partial _w = \partial _s, \end{aligned}$$

while the partial differential operators in Table 2 transform into

3.43a $$\begin{aligned} w \partial _w&= (u+1)\left( \frac{\partial u}{\partial u_0}\right) ^{-1}\partial _{u_0}\quad \text {and} \end{aligned}$$

3.43b $$\begin{aligned} z \partial _z&= (v+1)\left( \frac{\partial v}{\partial v_0}\right) ^{-1}\left[ -\left( \frac{\partial u}{\partial v_0}\right) \left( \frac{\partial u}{\partial u_0}\right) ^{-1}\partial _{u_0} + \partial _{v_0}\right] ; \end{aligned}$$

here, $u(s;u_0,v_0)$ and $v(s;v_0)$ are as given in (3.10). Thus, the mRNA autoactivation system (3.41) transforms to

3.44a $$\begin{aligned} \partial _s F^{(0)}_m&= - \kappa _0 F^{(0)}_m + \kappa _1 F^{(1)}_m - \alpha _M (u+1)\left( \frac{\partial u}{\partial u_0}\right) ^{-1}\partial _{u_0} F^{(0)}_{m-1}, \end{aligned}$$

3.44b $$\begin{aligned} \partial _s F^{(1)}_m&= \kappa _0 F^{(0)}_m - \kappa _1 F^{(1)}_m + \lambda \,u F^{(1)}_m + \alpha _M (u+1)\left( \frac{\partial u}{\partial u_0}\right) ^{-1}\partial _{u_0} F^{(0)}_{m-1}. \end{aligned}$$

To obtain explicit solutions to system (3.44), we adopt Assumption 3.1 and revert to the fast time scale $\sigma = \frac{s}{\varepsilon }$ to write the dynamical system (3.44) as a second-order ODE for $F^{(0)}_m$, which yields

3.45 $$\begin{aligned}&\left[ \partial _\sigma ^2 + \varepsilon (\kappa _0 + \kappa _1 - \lambda u)\partial _\sigma - \varepsilon ^2\kappa _0 \lambda u\right] F^{(0)}_m \nonumber \\&\quad = \big (\varepsilon ^2 \lambda u - \varepsilon \partial _\sigma \big ) \alpha _M (u+1)\left( \frac{\partial u}{\partial u_0}\right) ^{-1}\partial _{u_0} F^{(0)}_{m-1}. \end{aligned}$$

Using (3.44), we can express $F^{(1)}_m$ in terms of $F^{(0)}_m$ as

3.46 $$\begin{aligned} F^{(1)}_m = \frac{1}{\varepsilon \kappa _1} \left[ \partial _\sigma F^{(0)}_m + \varepsilon \kappa _0 F^{(0)}_m + \varepsilon \alpha _M (u+1)\left( \frac{\partial u}{\partial u_0}\right) ^{-1}\partial _{u_0} F^{(0)}_{m-1}\right] . \end{aligned}$$

To solve (3.45) (recursively), we expand $F^{(j)}_m$ ($j=0,1$) in powers of $\varepsilon$:

3.47 $$\begin{aligned} F^{(j)}_m(\sigma ) = \sum _{n=0}^\infty \varepsilon ^n F^{(j)}_{m,n}(\sigma ); \end{aligned}$$

recall Eq. (3.22). Together with the series expansion for *u* in (3.15), we thus obtain

3.48 $$\begin{aligned} \partial _\sigma ^2 F^{(0)}_{m,0}&= 0, \end{aligned}$$

3.49 $$\begin{aligned} \partial _\sigma ^2 F^{(0)}_{m,1}&= -(\kappa _0+\kappa _1 - \lambda \hat{u}_0)\partial _\sigma F^{(0)}_{m,0} - \partial _\sigma G_{m-1,0}, \end{aligned}$$

and

3.50 $$\begin{aligned}&\partial _\sigma ^2 F^{(0)}_{m,n} + (\kappa _0+\kappa _1 - \lambda \hat{u}_0) \partial _\sigma F^{(0)}_{m,n+1} -\lambda \sum _{k=0}^n \left( \hat{u}_{n+1-k} \partial _\sigma + \kappa _0\, \hat{u}_{n-k}\right) F^{(0)}_{m,k} \nonumber \\&\quad = -\partial _\sigma G^{(0)}_{m-1,n+1} + \lambda \sum _{k=0}^n \hat{u}_{n-k} G^{(0)}_{m-1,k} \end{aligned}$$

for $n \ge 2$; compare with Eqs. (3.23a), (3.23b), and (3.24). The coefficients $G^{(0)}_{m,n}$ in the above expression are defined from an expansion of the autoregulation term as

3.51 $$\begin{aligned} \alpha _M (u+1)\left( \frac{\partial u}{\partial u_0}\right) ^{-1}\partial _{u_0} F^{(0)}_{m-1} = \sum _{n=0}^\infty \varepsilon ^n G^{(0)}_{m-1,n}(u_0,\sigma ). \end{aligned}$$

From (3.46), we obtain

3.52 $$\begin{aligned} \partial _\sigma F^{(0)}_{m,0} = 0 \end{aligned}$$

and

3.53 $$\begin{aligned} F^{(1)}_{m,n} =\frac{1}{\kappa _1} \partial _\sigma F^{(0)}_{m,n+1} + \frac{\kappa _0}{\kappa _1} F^{(0)}_{m,n} + \frac{1}{\kappa _1} G^{(0)}_{m-1,n} \end{aligned}$$

for $n \ge 0$; recall (3.25) and (3.26). To solve Eqs. (3.48) through (3.53) iteratively, we fix *m*—the order of the expansion in $\delta$—and determine the solution to satisfactory order in *n*, the order of the expansion in $\varepsilon$. Then, we increase *m* to $m+1$ and take the result as input for the dynamics at order $m+1$. The resulting repeated iteration procedure yields an explicit expression for the generating functions $F^{(j)}$ ($j=0,1$) as double asymptotic series in both $\delta$ and $\varepsilon$.

The determination of appropriate initial conditions is largely analogous to the non-autoregulated case; see Sect. 3.4.1. However, with the inclusion of autoregulation into model B, we need to incorporate the possibility that $\chi (m_0,n_0)$ depends on the corresponding autoregulation rates. As in the case of model A, we expand $\chi (m_0,n_0)$ as a power series in $\delta$:

3.54 $$\begin{aligned} \chi (m_0,n_0) = \sum _{m=0}^\infty \delta ^m \chi _m(m_0,n_0); \end{aligned}$$

recall (2.51). In that case, the initial conditions for $F^{(j)}_{m,n}$ can be inferred from (3.32) to give

3.55a $$\begin{aligned} \left[ F^{(0)}_{m,0}\right] _{\sigma =0}&= \chi _m (1+u_0)^{m_0}(1+v_0)^{n_0} \quad \text {for all }m\ge 0, \end{aligned}$$

3.55b $$\begin{aligned} \left[ F^{(1)}_{0,0}\right] _{\sigma =0}&= (1-\chi _0) (1+u_0)^{m_0}(1+v_0)^{n_0}, \end{aligned}$$

3.55c $$\begin{aligned} \left[ F^{(1)}_{m,0}\right] _{\sigma =0}&= -\chi _m (1+u_0)^{m_0}(1+v_0)^{n_0} \quad \text {for all }m\ge 1,\text { and} \end{aligned}$$

3.55d $$\begin{aligned} \left[ F^{(j)}_{m,n}\right] _{\sigma =0}&= 0 \quad \text {for all } m\ge 0\text { and }n\ge 1,\text { with }j=0,1. \end{aligned}$$

Solutions to Eqs. (3.48)–(3.53) that incorporate the conditions in (3.55) for all types of autoregulation introduced in (3.2) can be found in Appendix B. Finally, our previous results on the inverse characteristic transformation in the non-autoregulated case from Sect. 3.4.2 can now be applied in a straightforward fashion to give solutions to the PDE system (3.6) with added autoregulation.

### Summary of main result

To summarise Sect. 3, we combine the analysis of the previous subsections to state our main result.

*Main result:* The PDE system (3.6) can be solved for sufficiently large values of $\gamma$; see Assumption 3.1. Its solutions $F^{(j)}(w,z,t)$ ($j=0,1$) are expressed as power series in the small parameter $\varepsilon =\frac{1}{\gamma }$; cf. (3.22). The coefficients $F_n^{(j)}(w,z,t)$ in these series, written in terms of the shifted variables *u* and *v* defined in (3.8), can be found by

1. solving recursively the second-order ODEs (3.23a) through (3.24) and using the identities in (3.25) and (3.26), incorporating the initial conditions in (3.32);
2. subsequently applying the inverse transformations in (3.36) and (3.37) to the resulting solutions;
3. and, finally, substituting $\sigma = \frac{t}{\varepsilon }$.

To illustrate the procedure described above, we state the resulting explicit expressions for the leading-order solution to (3.6) in terms of the original variables *w*, *z*, and *t* here:

3.56a $$\begin{aligned} F_0^{(0)}(w,z,t)&= \chi \big [1+(z-1)\mathrm{e}^{-t}\big ]^{n_0}\bigg \{\frac{1}{1+\mu (1-z)}+\mathrm{e}^{-[1+\mu (1-z)]\frac{t}{\varepsilon }}\nonumber \\&\quad \times \bigg [w - \frac{1}{1+\mu (1-z)}\bigg ]\bigg \}^{m_0}\quad \text {and} \end{aligned}$$

3.56b $$\begin{aligned} F_0^{(1)}(w,z,t)&= \frac{1-\chi }{\chi }F_0^{(0)}(w,z,t). \end{aligned}$$

Note that the sum $F_0^{(0)} + F_0^{(1)}$ corresponds precisely to the leading-order fast expansion found in Equation (21) of Bokes et al. (2012b).

If autoregulation as in (3.2) is incorporated into model B, the main result can be formulated as follows.

*Main result (autoregulatory extension):* The PDE system (3.6) incorporating any one type of autoregulation from Table 2 can be solved as long as $\gamma$ is sufficiently large and $\delta$ is sufficiently small; see Assumptions 3.1 and 3.7, respectively. Its solutions $F^{(j)}(w,z,t)$ ($j=0,1$) are expressed as double power series in the small parameters $\delta$ and $\varepsilon$, viz.

3.57 $$\begin{aligned} F^{(j)} = \sum _{m=0}^\infty \sum _{n=0}^\infty \delta ^m \varepsilon ^n F^{(j)}_{m,n}. \end{aligned}$$

The coefficients $F^{(j)}_{m,n}$ in these series, written in terms of the shifted variables *u* and *v* defined in (3.8), can be found by

- solving recursively the second-order ODEs (3.48) through (3.50) for fixed *m* and using the identities in (3.52) and (3.53), incorporating the initial conditions in (3.55);
- increasing *m* to $m+1$, and repeating step (1a) until a sufficient accuracy in $\delta$ (and $\varepsilon$) is attained;
- subsequently applying the inverse transformations in (3.36) and (3.37) to the resulting solutions;
- and, finally, substituting $\sigma = \frac{t}{\varepsilon }$.

## From generating function to propagator

The final step in our analytical method consists in reconstructing the probabilities $P^{(j)}_n$ (model A) and $P^{(j)}_{m,n}$ (model B), respectively, from the explicit expressions for the associated generating functions $F^{(j)}$ ($j=0,1$), which were the main analytical outcome of Sects. 2 and 3.

In principle, the relation between probabilities and probability-generating functions is clear from the definition of the latter, and is given in (2.6) and (3.5), respectively. Specifically, probabilities can be expressed in terms of derivatives of their generating functions as follows:

4.1a $$\begin{aligned} P^{(j)}_n(t)&= \frac{1}{n!}\left[ \frac{\partial ^n}{\partial z^n}F^{(j)}(z,t)\right] _{z=0}&\text {(model A)}, \end{aligned}$$

4.1b $$\begin{aligned} P^{(j)}_{m,n}(t)&= \frac{1}{m! n!}\left[ \frac{\partial ^{m+n}}{\partial w^m \partial z^n}F^{(j)}(w,z,t)\right] _{(w,z)=(0,0)}&\text {(model B)}. \end{aligned}$$

However, explicit expressions for the *n*th and $(m+n)$th order derivatives, respectively, of these generating functions become progressively unwieldy with increasing *m* and *n*. Indeed, from the expressions for the generating functions $F^{(j)}$ obtained previously, which combine (2.26) for specific initial conditions as in (2.54) with the inverse characteristic transformation in (2.56), it is clear that finding explicit expressions for derivatives of arbitrary order is very difficult indeed, if it is possible at all.^1^

To complete successfully the final step towards approximating propagators for parameter inference in the present setting, we abandon the requirement of deriving explicit expressions for the probabilities $P^{(j)}_n$ and $P^{(j)}_{m,n}$. Instead, we use the standard Cauchy integral formula for derivatives of holomorphic functions to write

4.2a $$\begin{aligned} P^{(j)}_n(t)&= \frac{1}{2\pi i} \oint _{\gamma _A} \frac{F^{(j)}(z,t)}{z^{n+1}}\,\text {d}z&\text {(model A)}, \end{aligned}$$

4.2b $$\begin{aligned} P^{(j)}_{m,n}(t)&= \frac{1}{(2\pi i)^2} \oiint \nolimits _{\gamma _B} \frac{F^{(j)}(w,z,t)}{w^{m+1}z^{n+1}}\,\text {d}w\text {d}z&\text {(model B)}; \end{aligned}$$

here, $\gamma _A$ is a suitably chosen contour around $z=0$, while $\gamma _B$ is a (double) contour around $(w,z) = (0,0)$.

The above expression of probabilities as integrals is well suited for an efficient numerical implementation, which is naturally incorporated into the realisation of the parameter inference scheme discussed in Sect. 1. From a numerical perspective, the integral formula in (4.2) has the additional advantage that the values of $F^{(j)}$ on (a discretisation of) the integral contours $\gamma _{A}$ and $\gamma _{B}$, respectively, only have to be determined once to yield propagators for any values of *m* and *n* and fixed initial states $m_0$ and $n_0$. Moreover, we are free to choose the integration contours $\gamma _{A}$ and $\gamma _{B}$, which allows us to accelerate the calculation of these integrals; see Bornemann (2011). Here, we note that the choice of circular integration contours with unit radius, and subsequent discretisation of those contours as *M*-sided and *N*-sided polygons, respectively, coincides with the ‘Fourier mode’ approach, as presented by Bokes et al. (2012a).

### Remark 4.1

By introducing the Cauchy integral formula for derivatives of holomorphic functions in (4.2), we implicitly assume that the integration contours $\gamma _{A}$ and $\gamma _{B}$ are chosen such that they lie completely within the open neighbourhoods of the origin in $\mathbb {C}$ and $\mathbb {C}^2$, respectively, where the canonical complex extensions of the generating functions $F^{(j)}$—which exist by the Cauchy-Kowalevski theorem—are holomorphic. In other words, $\gamma _{A}$ and $\gamma _{B}$ must be chosen such that any poles of $F^{(j)}$ lie outside of these integration contours. The expansion for $u_0$ in (3.36) shows that this is not a moot point: the generating functions $F^{(j)}$ resulting for model B, as established in Sect. 3, will generically have a pole at $v = \frac{1}{\mu }$, i.e. at $z = 1 +\frac{1}{\mu }$. As $\mu$ is positive, by (3.4), choosing the *z*-contour of $\gamma _B$ to be a circle with at most unit radius allows us to avoid that pole.

### Incorporation of ${\pmb \chi }$

In the course of the analysis presented in Sects. 2 and 3, the introduction of the parameter $\chi$ was necessary to obtain definite, explicit expressions for the generating functions as solutions to the PDE systems (2.7), (2.8), and (3.6); see Definitions 2.4 and 3.4. The successful implementation of these expressions in a parameter inference scheme requires us to decide how to incorporate that new parameter. We identify three options here.

1. Before implementing parameter inference, we can marginalise over the new parameter $\chi$ to eliminate it altogether, using a predetermined measure $\text {d} \mu (\chi )$, which adds an additional integration step to the requisite numerical scheme.
2. We can make a choice for $\chi$ that is based on the specifics of the model under consideration. Thus, exploiting the Markov property of the stochastic models underlying (2.3), (2.5), and (3.3), we may use the switching rates and any autoregulation rates to express $\chi$ in model A as
  4.3a $$\begin{aligned} \chi (n_0)&= \frac{c_b}{c_b+c_f+a n_0}&\text {(autoactivation)}, \end{aligned}$$
  4.3b $$\begin{aligned} \chi (n_0)&= \frac{c_b+r n_0}{c_b+r n_0+c_f}&\text {(autorepression)}; \end{aligned}$$
  the corresponding expressions for model B read
  4.4a $$\begin{aligned} \chi (m_0,n_0)&= \frac{k_1}{k_0 + k_1}&\text {(no autoregulation)}, \end{aligned}$$
  4.4b $$\begin{aligned} \chi (m_0,n_0)&= \frac{k_1}{k_0 + k_1 + a_M m_0}&\text {(mRNA autoactivation)}, \end{aligned}$$
  4.4c $$\begin{aligned} \chi (m_0,n_0)&= \frac{k_1 + r_M m_0}{k_0 + k_1 + r_M m_0}&\text {(mRNA autorepression)}, \end{aligned}$$
  4.4d $$\begin{aligned} \chi (m_0,n_0)&= \frac{k_1}{k_0 + k_1 + a_P n_0}&\text {(protein autoactivation)}, \end{aligned}$$
  4.4e $$\begin{aligned} \chi (m_0,n_0)&= \frac{k_1 + r_P n_0}{k_0 + k_1 + r_P n_0}&\text {(protein autorepression)}. \end{aligned}$$
3. We can determine $\chi$ ‘experimentally’ by including the latter in the parameter set that is to be inferred in the (numerical) process of parameter inference.

Note that step 2 has been anticipated in the analysis of model A, by introducing the series expansion in (2.51). Indeed, by Assumption 2.2, we can expand $\chi (n_0)$ as

4.5a $$\begin{aligned} \chi&= \frac{\kappa _b}{\kappa _b+\kappa _f+\delta \alpha n_0} = \frac{\kappa _b}{\kappa _f + \kappa _b} \sum _{m=0}^\infty \delta ^m \left( \frac{-\alpha n_0}{\kappa _f+\kappa _b}\right) ^m&\text {(autoactivation)}, \end{aligned}$$

4.5b $$\begin{aligned} \chi&= \frac{\kappa _b + \delta \rho n_0}{\kappa _b+\kappa _f+\delta \rho n_0} \nonumber \\&= \frac{\kappa _b}{\kappa _f + \kappa _b} - \frac{\kappa _f}{\kappa _f+\kappa _b}\sum _{m=1}^\infty \delta ^m \left( \frac{-\rho n_0}{\kappa _f+\kappa _b}\right) ^m&\text {(autorepression)}. \end{aligned}$$

Likewise, when autoregulation is added to model B, Assumption 3.7 implies an expansion for $\chi (m_0,n_0)$ of the form

4.6a $$\begin{aligned} \chi&= \frac{\kappa _1}{\kappa _0 + \kappa _1 + \delta \alpha _M m_0}\nonumber \\&= \frac{\kappa _1}{\kappa _0 + \kappa _1} \sum _{m=0}^\infty \delta ^m \left( \frac{-\alpha _M m_0}{\kappa _0+\kappa _1}\right) ^m&\text {(mRNA autoactivation)}, \end{aligned}$$

4.6b $$\begin{aligned} \chi&= \frac{\kappa _1 + \delta \rho _M m_0}{\kappa _0 + \kappa _1 + \delta \rho _M m_0} \nonumber \\&= \frac{\kappa _1}{\kappa _0 + \kappa _1} - \frac{\kappa _0}{\kappa _0+\kappa _1}\sum _{m=1}^\infty \delta ^m \left( \frac{-\rho _M m_0}{\kappa _0+\kappa _1}\right) ^m&\text {(mRNA autorepression)}, \end{aligned}$$

4.6c $$\begin{aligned} \chi&= \frac{\kappa _1}{\kappa _0 + \kappa _1 + \delta \alpha _P n_0} \nonumber \\&= \frac{\kappa _1}{\kappa _0 + \kappa _1} \sum _{m=0}^\infty \delta ^m \left( \frac{-\alpha _P n_0}{\kappa _0+\kappa _1}\right) ^m&\text {(protein autoactivation)}, \end{aligned}$$

4.6d $$\begin{aligned} \chi&= \frac{\kappa _1 + \delta \rho _P n_0}{\kappa _0 + \kappa _1 + \delta \rho _P n_0} \nonumber \\&= \frac{\kappa _1}{\kappa _0 + \kappa _1} - \frac{\kappa _0}{\kappa _0+\kappa _1}\sum _{m=1}^\infty \delta ^m \left( \frac{-\rho _P n_0}{\kappa _0+\kappa _1}\right) ^m&\text {(protein autorepression)}. \end{aligned}$$

## Discussion and outlook

In the present article, we have developed an analytical method for obtaining explicit, fully time-dependent expressions for the probability-generating functions that are associated to models for stochastic gene expression. Moreover, we have presented a computationally efficient approach which allows us to derive model predictions (in the form of propagators) from these generating functions, using the Cauchy integral formula. It is important to note that our method does not make any steady-state or long-evolution-time approximations. On the contrary, the perturbative nature of our approach naturally optimises its applicability over relatively short (or intermediate) time scales; see also Remark 3.6. As is argued in Sect. 1.1, such relatively short evolution times naturally occur in the calculation of quantities such as the log-likelihood, as defined in Eq. (1.1). Therefore, our analytical approach is naturally suited to an implementation in a Bayesian parameter inference scheme, such as is outlined in Sect. 1.1.

As mentioned in Sects. 2.2 and 3.3, the introduction of Assumptions 2.2 and 3.7 in our analysis of the systems of PDEs and ODEs that are obtained via the generating function approach is *necessary* for determining explicit expressions for the generating functions themselves. Therefore, we can only be certain of the validity of our approach if we assume that the autoregulation rates are small in comparison with other model parameters, as is done there. Moreover, in the analysis of model B, we have to assume that the protein decay rate is smaller than the decay rate of mRNA; recall Assumption 3.1. That assumption is valid for a large class of (microbial) organisms (Shahrezaei and Swain 2008a; Yu et al. 2006); however, it is by no means generic, as the two decay rates are often comparable in mammalian cells (Schwanhäusser et al. 2011; Vogel and Marcotte 2012). Since the accuracy of approximation of the explicit expressions for the generating functions derived here is quantified in terms of orders of the perturbation parameter(s), see e.g. Remark 3.6, violation of Assumption 2.2, 3.1, or 3.7 will decrease the predictive power of the results obtained by the application of the analytical method developed in the article.

The method which is described in Sect. 1.2, and outlined visually in Fig. 5, hence provides a generic framework for the analysis of stochastic gene expression models such as model A (Fig. 3 and Sect. 2.1) and model B (Fig. 4 and Sect. 3.1). Note that, for example, the steady-state and long-evolution-time approximations derived by Shahrezaei and Swain (2008a) could be extended to autoregulatory systems via the same approach. However, as is apparent from the (differences between the) analysis presented in Sects. 2 and 3, the ‘path to an explicit solution’ is highly model-dependent. The decision on which analytical techniques to apply, such as the perturbative expansion postulated in (3.22), has to be made on a case-by-case basis. The success of the method presented in the article fully depends on whether the resulting dynamical systems can be solved *explicitly*. To that end, it is highly beneficial that the systems (2.19) and (3.19) obtained here are linear, which is a direct consequence of the fact that all reactions described in the reaction schemes in (2.1) and (2.2), as well as in (3.1) and (3.2), are of *first order*. Inclusion of second-order reactions would introduce both nonlinear terms and second-order differential operators in the PDE systems for the corresponding generating functions, which would severely increase the complexity of these systems, thus preventing us from obtaining explicit solutions.

The method presented in this article, and the results thus obtained, can be seen, first and foremost, as the natural extension of previous work by Popović et al. (2016). Analytical results for the classes of models studied here can be found in several earlier articles. We mention the article by Shahrezaei and Swain (2008a), where a leading-order approximation was obtained in a long-evolution-time and steady-state limit. Bokes et al. (2012a) derived analytical expressions for stationary distributions in a model that is equivalent to that considered by Popović et al. (2016). Also for that model, a time-scale separation was exploited by Bokes et al. (2012b), in a manner that is similar to the present article, to obtain leading-order analytical expressions on both time scales. The model that is referred to as Model A in Sect. 2 was analysed in a steady-state setting by Iyer-Biswas and Jayaprakash (2014) via the Poisson integral transform. A similar model was studied by Hornos et al. (2005), were a generating function approach was used; making a steady-state Ansatz, the authors were able to obtain an explicit solution for the generating function in terms of special (Kummer) functions; see also NIST Digital Library of Mathematical Functions . The same model was later solved in a fully time-dependent context by Ramos et al. (2011), after a cleverly chosen variable substitution, in terms of another class of special (Heun) functions; cf. again NIST Digital Library of Mathematical Functions .

Other authors have attempted to solve several classes of CMEs directly, i.e. without resorting to generating function techniques or integral transforms. A noteworthy example is the work of Jahnke and Huisinga (2007) on monomolecular systems. Another, more recent example can be found in the work by Iserles and MacNamara (2017), where exact solutions are determined for explicitly time-dependent isomerisation models.

It is important to emphasise that the ‘time dependence’ referred to in the title of the present article is solely due to the dynamic nature of the underlying stochastic process, and that it hence manifests exclusively through time derivatives in the associated CMEs, such as e.g. in (2.3). In particular, none of the model parameters are time-dependent, as opposed to, for example, the system studied by Iserles and MacNamara (2017). The inclusion of such explicitly time-dependent parameters would be a starting point for incorporating the influence of (extrinsic) noise in the context of the model categories considered in the article.

The availability of analytical expressions for generating functions does, in principle, allow one to try to obtain insight into the underlying processes by studying the explicit form of said expressions, as has been done e.g. by Bokes et al. (Bokes et al. 2012a, b). However, the complex nature of the processes we analyse here seems to preclude such insights. For example, the integrals over confluent hypergeometric functions, which appear in (2.29), cannot themselves be efficiently expressed in terms of (other) special functions. Still, that complication does not necessarily pose an obstacle to the application we ultimately have in mind, i.e. to Bayesian parameter inference. As the last step in our method—the extraction of propagators from generating functions, see Sect. 4—is numerical, the precise functional form of the generating function is not of importance. The mere fact that an explicit expression can be obtained is sufficient for the application of the Cauchy integral formula, where these generating functions enter into the calculation of the appropriate integrals; see again Sect. 4.

The analytical approach explored in the article does not, of course, represent the only feasible way of obtaining numerical values for propagator probabilities, which can, in turn, serve as input for a Bayesian parameter inference scheme. For an example of a direct numerical method in which the Cauchy integral plays a central role, the reader is referred to the work by MacNamara (2015). Our main motivation for pursuing an analytical alternative is reducing the need for potentially lengthy numerical simulations. An efficient implementation of the resulting expressions can result in (significantly) reduced computation times; see, for example, the work by Bornemann (2011). The optimisation of the underlying numerical procedures is, however, beyond the scope of the present article in particular, and of our research programme in general.

The analytical results obtained thus far, as presented in the article, are ready for implementation in a Bayesian parameter inference framework. An analysis of the performance of the resulting approximations to the associated generating functions in the spirit of the article by Feigelman et al. (2015), where parameter inference is tested on simulated data based on specific stochastic models, is ongoing work. Moreover, the successful application of our analytical method to specific model categories, such as are represented by model A and model B, suggests several feasible expansions of the ‘model library’ for which explicit expressions for the corresponding generating functions can be constructed. Thus, stochastic models comprised of multiple proteins represent a natural next stage, bringing the analysis of toggle switch-type models within reach. In addition, one could begin exploring the vast field of gene regulatory networks by considering a simple two-protein system with, for example, activator-inhibitor interaction. Under the assumption of small interaction rates, the resulting PDE system for the associated generating function would be directly amenable to the analytical method described in the article. The analysis of these and similar systems could be a topic for future research.

## Model A: explicit expressions

An explicit expression for (2.27) can be stated as follows:

A.1 $$\begin{aligned} F^{(1)}_0(w)&= \frac{1}{\kappa _b}\left( w \frac{\text {d}}{\text {d}w}F^{(0)}_0 + \kappa _f F^{(0)}_0\right) \nonumber \\&= c_1 \frac{\kappa _f}{\kappa _b} \left( {}_1 F_1(\kappa _f,1+\kappa _f+\kappa _b,w) \right. \nonumber \\&\quad \left. + \frac{w}{1+\kappa _f+\kappa _b}{}_1 F_1(1+\kappa _f,2+\kappa _f+\kappa _b,w) \right) \nonumber \\&\quad - c_2 w^{-\kappa _f-\kappa _b} \left( {}_1 F_1(-\kappa _b,1-\kappa _f-\kappa _b,w)\right. \nonumber \\&\quad \left. +\frac{w}{1-\kappa _f-\kappa _b}{}_1 F_1(1-\kappa _b,2-\kappa _f-\kappa _b,w)\right) , \end{aligned}$$

which can be shown to be equal to (2.35) using §13.3(i) of NIST Digital Library of Mathematical Functions when $\hat{c}_1 = c_1 \frac{\kappa _f}{\kappa _b}$ and $\hat{c}_2 = c_2$. Explicit expressions involving the integration limits $c_{3}$ and $c_{4}$ in (2.29), respectively, are obtained as

A.2 $$\begin{aligned}&\int _{c_3}^{\lambda v_0} \!\!\!{}_1 F_1(-\kappa _b,1-\kappa _f-\kappa _b,\hat{w}) \, g(\hat{w}) \;\text {d} \hat{w} \nonumber \\&\quad = \mathrm{e}^{-\lambda v_0} \Bigg \{\chi _1 (1+v_0)^{n_0} \frac{\lambda v_0 \kappa _b}{1-\kappa _f-\kappa _b} {}_1 F_1(1-\kappa _b,2-\kappa _f-\kappa _b,\lambda v_0) \nonumber \\&\qquad - {}_1F_1(-\kappa _b,1-\kappa _f-\kappa _b,\lambda v_0) \left[ \alpha (w+\lambda )\left( \partial _w + \frac{v_0}{w}\partial _{v_0}\right) F^{(0)}_0\right] _{w=\lambda v_0}\Bigg \}\qquad \qquad \end{aligned}$$

and

A.3 $$\begin{aligned}&\int _{c_4}^{\lambda v_0} \!\!\!{}_1 F_1(\kappa _f,1+\kappa _f+\kappa _b,\hat{w})\, \frac{g(\hat{w})}{\hat{w}^{-\kappa _f-\kappa _b}} \;\text {d} \hat{w}\nonumber \\&\quad = -\mathrm{e}^{-\lambda v_0}(\lambda v_0)^{\kappa _f+\kappa _b} \Bigg \{\chi _1(1+v_0)^{n_0}(\kappa _f+\kappa _b) {}_1 F_1(\kappa _f,1+\kappa _f+\kappa _b,\lambda v_0) \nonumber \\&\qquad + \chi _1(1+v_0)^{n_0} \frac{\lambda v_0 \kappa _f}{1+\kappa _f+\kappa _b} {}_1F_1(1+\kappa _f,2+\kappa _f+\kappa _b,\lambda v_0) \nonumber \\&\qquad + {}_1 F_1(\kappa _f,1+\kappa _f+\kappa _b,\lambda v_0)\left[ \alpha (w+\lambda )\left( \partial _w + \frac{v_0}{w}\partial _{v_0}\right) F^{(0)}_0\right] _{w=\lambda v_0}\Bigg \},\qquad \qquad \end{aligned}$$

respectively, with $F^{(0)}_0$ as in (2.26). Similarly, explicit expression that involve the integration limits $\hat{c}_{3}$ and $\hat{c}_{4}$ in (2.38), respectively, read

A.4 $$\begin{aligned}&\int _{\hat{c}_3}^{\lambda v_0} \!\!\!{}_1 F_1(1-\kappa _b,1-\kappa _f-\kappa _b,\hat{w}) \, h(\hat{w}) \;\text {d} \hat{w} \nonumber \\&\quad = -\mathrm{e}^{-\lambda v_0} \Bigg \{\chi _1(1+v_0)^{n_0} \lambda v_0\, {}_1F_1(1-\kappa _b,1-\kappa _f-\kappa _b,\lambda v_0) \nonumber \\&\qquad -\chi _1 (1+v_0)^{n_0} \frac{\lambda v_0 (1-\kappa _b)}{1-\kappa _f-\kappa _b} {}_1 F_1(2-\kappa _b,2-\kappa _f-\kappa _b,\lambda v_0) \nonumber \\&\qquad + {}_1F_1(1-\kappa _b,1{-}\kappa _f-\kappa _b,\lambda v_0) \left[ \rho (w{+}\lambda )\left( \partial _w {+} \frac{v_0}{w}\partial _{v_0}\right) F^{(1)}_0\right] _{w=\lambda v_0}\Bigg \}\qquad \qquad \end{aligned}$$

and

A.5 $$\begin{aligned}&\int _{\hat{c}_4}^{\lambda v_0} \!\!\!{}_1 F_1(\kappa _f,1+\kappa _f+\kappa _b,\hat{w})\, \frac{h(\hat{w})}{\hat{w}^{-\kappa _f-\kappa _b}} \;\text {d} \hat{w}\nonumber \\&\quad = \mathrm{e}^{-\lambda v_0}(\lambda v_0)^{\kappa _f+\kappa _b} \Bigg \{\chi _1(1+v_0)^{n_0}(\kappa _f+\kappa _b-\lambda v_0) {}_1 F_1(1+\kappa _f,1+\kappa _f+\kappa _b,\lambda v_0) \nonumber \\&\qquad + \chi _1(1+v_0)^{n_0} \frac{\lambda v_0 (1+\kappa _f)}{1+\kappa _f+\kappa _b} {}_1F_1(2+\kappa _f,2+\kappa _f+\kappa _b,\lambda v_0) \nonumber \\&\qquad + {}_1 F_1(1+\kappa _f,1+\kappa _f+\kappa _b,\lambda v_0)\left[ \rho (w+\lambda )\left( \partial _w + \frac{v_0}{w}\partial _{v_0}\right) F^{(1)}_0\right] _{w=\lambda v_0}\Bigg \},\nonumber \\ \end{aligned}$$

respectively, with $F^{(1)}_0$ as in (2.35).

## Model B with autoregulation: explicit expressions

To leading order in $\delta$, i.e. for $m=0$, the right-hand side of Eq. (3.45) vanishes; hence, the expressions in Eqs. (3.20) and (3.21) apply. In other words, we can identify $F^{(j)}_{0,n}$ with $F^{(j)}_n$ as given in (3.27) and (3.28), for $j=0,1$ and $n= 0,1,2$. The free constants $f_i$ and $g_i$ are as stated in (3.33), with $\chi$ replaced with $\chi _0$. In the following sections, we present explicit expressions for the first-order correction in $\delta$—i.e. for $m=1$—for all autoregulatory scenarios listed in (3.2).

### mRNA autoactivation

We can solve (3.45) to second order in $\varepsilon$, i.e. for $n=2$, applying the initial conditions in (3.55) to obtain

B.1 $$\begin{aligned} F^{(0)}_{1,0}&= h_{0,M}, \end{aligned}$$

B.2 $$\begin{aligned} F^{(0)}_{1,1}&= h_{1,M} + j_{1,M}\sigma + \alpha _M \chi _0 m_0 (1+u_0)^{m_0-1}(1+v_0)^{n_0} \frac{\mathrm{e}^{-(1-\mu v_0)\sigma }}{(1-\mu v_0)^2},\quad \text {and} \end{aligned}$$

B.3 $$\begin{aligned} F^{(0)}_{1,2}&= h_{2,M} + j_{2,M}\sigma \end{aligned}$$

B.4 $$\begin{aligned}&\quad + \alpha _M m_0 (1+u_0)^{m_0-1}(1+v_0)^{n_0} \kappa _1 \left[ \frac{\mathrm{e}^{-(1-\mu v_0)\sigma }}{1-\mu v_0}\left( \frac{1}{1-\mu v_0} + \sigma \right) \right. \nonumber \\&\quad \left. - \frac{\sigma ^2}{2}(1-\mu v_0) \left( u_0 - \frac{\mu v_0}{1-\mu v_0}\right) \right] \nonumber \\&\quad + \alpha _M m_0 (1+u_0)^{m_0-1}(1+v_0)^{n_0} \chi _0 \Bigg \{\frac{\mathrm{e}^{-(1-\mu v_0)\sigma }}{1-\mu v_0}\left[ \frac{3 \mu v_0}{(1-\mu v_0)^2}\right. \nonumber \\&\quad \left. - \sigma \left( \kappa _0+\kappa _1 - \frac{2 \mu v_0}{1-\mu v_0}\right) + \frac{\sigma ^2}{2} \mu v_0\right] \nonumber \\&\quad +\sigma ^2 (\kappa _0+\kappa _1)(1-\mu v_0) \left( u_0 - \frac{\mu v_0}{1-\mu v_0}\right) \Bigg \} \nonumber \\&\quad - (1+u_0)^{m_0}(1+v_0)^{n_0} \chi _1 \left[ \frac{\lambda \kappa _1 \mathrm{e}^{(1-\mu v_0)\sigma }}{(1-\mu v_0)^2} \left( u_0 - \frac{\mu v_0}{1-\mu v_0}\right) \right. \nonumber \\&\quad \left. + \frac{\sigma ^2}{2}\left( \frac{\lambda \kappa _1 \mu v_0}{1-\mu v_0}-(\kappa _0+\kappa _1)^2\right) \right] , \end{aligned}$$

which yields

B.5 $$\begin{aligned} F^{(1)}_{1,0}&= \frac{1}{\kappa _1} \left[ j_{1,M} + \kappa _0 h_{0,M} +\alpha _M m_0 \chi _0 (1+u_0)^{m_0-1}(1+v_0)^{n_0} \left( u_0 - \frac{\mu v_0}{1-\mu v_0}\right) \right] \nonumber \\&\quad \quad \text {and} \end{aligned}$$

B.6 $$\begin{aligned} F^{(1)}_{1,1}&= \frac{j_{2,M} + \kappa _0(h_{1,M} + j_{1,M} \sigma )}{\kappa _1} \nonumber \\&\quad - \frac{\chi _1}{\kappa _1} (1+u_0)^{m_0}(1+v_0)^{n_0}\left[ \frac{\lambda \kappa _1 \mathrm{e}^{(1-\mu v_0)\sigma }}{1-\mu v_0} \left( u_0 - \frac{\mu v_0}{1-\mu v_0}\right) \right. \nonumber \\&\quad \left. + \sigma \left( \frac{\lambda \kappa _1 \mu v_0}{1-\mu v_0} - (\kappa _0+\kappa _1)^2\right) \right] \nonumber \\&\quad - \alpha _M \frac{\chi _0}{\kappa _1} m_0 (1+u_0)^{m_0-1}(1+v_0)^{n_0} \left[ \frac{\mu v_0}{(1-\mu v_0)^3} + \frac{\kappa _1 \mathrm{e}^{-(1-\mu v_0)\sigma }}{(1-\mu v_0)^2}\right. \nonumber \\&\quad \left. - \sigma (\kappa _0+\kappa _1)\left( u_0 - \frac{\mu v_0}{1-\mu v_0}\right) \right] , \end{aligned}$$

where

B.7 $$\begin{aligned} h_{0,M}&= \chi _1 (1+u_0)^{m_0}(1+v_0)^{n_0}, \end{aligned}$$

B.8 $$\begin{aligned} h_{1,M}&= -\alpha _M \chi _0 m_0 (1+u_0)^{m_0-1}(1+v_0)^{n_0}\frac{1}{(1-\mu v_0)^2}, \end{aligned}$$

B.9 $$\begin{aligned} j_{1,M}&= -\chi _1(\kappa _0+\kappa _1)(1+u_0)^{m_0}(1+v_0)^{n_0} \nonumber \\&\quad + \alpha _M \chi _0 m_0 (1+u_0)^{m_0-1}(1+v_0)^{n_0} \left( u_0 - \frac{\mu v_0}{1-\mu v_0}\right) , \end{aligned}$$

B.10 $$\begin{aligned} h_{2,M}&= \chi _1 (1+u_0)^{m_0}(1+v_0)^{n_0} \frac{\lambda \kappa _1}{(1-\mu v_0)^2}\left( u_0-\frac{\mu v_0}{1-\mu v_0}\right) \nonumber \\&\quad - \alpha _M m_0 (1+u_0)^{m_0-1}(1+v_0)^{n_0} \frac{1}{(1-\mu v_0)^3} \left( \kappa _1 + \frac{3 \chi _0 \mu v_0}{1-\mu v_0}\right) ,\quad \text {and} \end{aligned}$$

B.11 $$\begin{aligned} j_{2,M}&= \chi _1 (1+u_0)^{m_0}(1+v_0)^{n_0} \frac{\lambda \kappa _1}{1-\mu v_0} \left( u_0-\frac{\mu v_0}{1-\mu v_0}\right) \nonumber \\&\quad + \alpha _M \chi _0 m_0 (1+u_0)^{m_0-1}(1+v_0)^{n_0} \frac{1}{(1-\mu v_0)^2}\left( \kappa _0 + \kappa _1 + \frac{\mu v_0}{1-\mu v_0}\right) . \end{aligned}$$

### mRNA autorepression

For the case of mRNA autorepression, we replace $\alpha _M (u+1)\left( \frac{\partial u}{\partial u_0}\right) ^{-1}\partial _{u_0} F^{(0)}_{m-1}$ in Eqs. (3.45) and (3.46) with

B.12 $$\begin{aligned} -\rho _M (u+1)\left( \frac{\partial u}{\partial u_0}\right) ^{-1}\partial _{u_0} F^{(1)}_{m-1}, \end{aligned}$$

which yields

B.13 $$\begin{aligned} F^{(0)}_{1,0}&= k_{0,M}, \end{aligned}$$

B.14 $$\begin{aligned} F^{(0)}_{1,1}&= k_{1,M} + l_{1,M}\sigma - \rho _M (1-\chi _0) m_0 (1+u_0)^{m_0-1}(1+v_0)^{n_0} \frac{\mathrm{e}^{-(1-\mu v_0)\sigma }}{(1-\mu v_0)^2},\quad \text {and} \end{aligned}$$

B.15 $$\begin{aligned} F^{(0)}_{1,2}&= k_{2,M} + l_{2,M}\sigma \nonumber \\&\quad - \rho _M m_0 (1+u_0)^{m_0-1}(1+v_0)^{n_0} \kappa _0 \left[ \frac{\mathrm{e}^{-(1-\mu v_0)\sigma }}{(1-\mu v_0)^2}\left( \frac{1}{1-\mu v_0} + \sigma \right) \right. \nonumber \\&\quad \left. - \frac{\sigma ^2}{2}\left( u_0 - \frac{\mu v_0}{1-\mu v_0}\right) \right] \nonumber \\&\quad + \rho _M m_0 (1+u_0)^{m_0-1}(1+v_0)^{n_0} (1-\chi _0) \Bigg \{\frac{\lambda \mathrm{e}^{(1-\mu v_0)\sigma }}{(1-\mu v_0)^2}\left( u_0 - \frac{\mu v_0}{1-\mu v_0}\right) ^2 \nonumber \\&\quad + \frac{\lambda \mathrm{e}^{-(1-\mu v_0)\sigma }}{(1-\mu v_0)^3}\left( u_0 - \frac{\mu v_0}{1-\mu v_0}\right) - \frac{(\lambda +3)\mu v_0 \mathrm{e}^{-(1-\mu v_0)\sigma }}{(1-\mu v_0)^4}\nonumber \\&\quad - \sigma \frac{\mathrm{e}^{-(1-\mu v_0)\sigma }}{(1-\mu v_0)^2}\left[ \frac{(\lambda + 2)\mu v_0}{1-\mu v_0} - (\kappa _0+\kappa _1)\right] \nonumber \\&\quad - \frac{\sigma ^2}{2} \left[ \frac{\mu v_0 \mathrm{e}^{-(1-\mu v_0)\sigma }}{(1-\mu v_0)^2} + \left( u_0-\frac{\mu v_0}{1-\mu v_0}\right) \left( 2(\kappa _0+\kappa _1) - \frac{\lambda \mu v_0}{1-\mu v_0}\right) \right] \Bigg \} \nonumber \\&\quad - (1+u_0)^{m_0}(1+v_0)^{n_0} \chi _1 \left[ \frac{\lambda \kappa _1 \mathrm{e}^{(1-\mu v_0)\sigma }}{(1-\mu v_0)^2} \left( u_0 - \frac{\mu v_0}{1-\mu v_0}\right) \right. \nonumber \\&\quad \left. + \frac{\sigma ^2}{2}\left( \frac{\lambda \kappa _1 \mu v_0}{1-\mu v_0}-(\kappa _0+\kappa _1)^2\right) \right] \nonumber \\&\quad + \rho _M (1+u_0)^{m_0}(1+v_0)^{n_0}(1-\chi _0) \frac{\lambda }{(1-\mu v_0)^2} \nonumber \\&\quad \times \left[ \mathrm{e}^{(1-\mu v_0)\sigma }\left( u_0 - \frac{\mu v_0}{1-\mu v_0}\right) + \frac{\mathrm{e}^{-(1-\mu v_0)\sigma }}{1-\mu v_0}\right] . \end{aligned}$$

Hence, it follows that

B.16 $$\begin{aligned} F^{(1)}_{1,0}&= \frac{1}{\kappa _1} \left[ l_{1_M} {+} \kappa _0 k_{0,M} {-}\rho _M m_0 (1{-}\chi _0) (1{+}u_0)^{m_0-1}(1{+}v_0)^{n_0} \left( u_0 - \frac{\mu v_0}{1-\mu v_0}\right) \right] \nonumber \\&\quad \quad \text {and} \end{aligned}$$

B.17 $$\begin{aligned} F^{(1)}_{1,1}&= \frac{l_{2,M} + \kappa _0(k_{1,M} + l_{1,M} \sigma )}{\kappa _1}\nonumber \\&\quad - \frac{\chi _1}{\kappa _1} (1+u_0)^{m_0}(1+v_0)^{n_0}\left[ \frac{\lambda \kappa _1 \mathrm{e}^{(1-\mu v_0)\sigma }}{1-\mu v_0} \left( u_0 - \frac{\mu v_0}{1-\mu v_0}\right) \right. \nonumber \\&\quad \left. + \sigma \left( \frac{\lambda \kappa _1 \mu v_0}{1-\mu v_0} - (\kappa _0+\kappa _1)^2\right) \right] \nonumber \\&\quad + \rho _M (1+u_0)^{m_0}(1+v_0)^{n_0} (1-\chi _0)\frac{\lambda }{\kappa _1(1-\mu v_0)}\nonumber \\&\quad \times \left[ \left( u_0 - \frac{\mu v_0}{1-\mu v_0}\right) -\frac{1}{1-\mu v_0}\right] \nonumber \\&\quad + \rho _M (1-\chi _0) m_0 (1+u_0)^{m_0-1}(1+v_0)^{n_0} \Bigg [\frac{\mathrm{e}^{-(1-\mu v_0)\sigma }}{(1-\mu v_0)^2} \nonumber \\&\quad - \frac{\lambda }{\kappa _1(1-\mu v_0)^2}\left( u_0-\frac{\mu v_0}{1-\mu v_0}\right) \nonumber \\&\quad + \frac{\mu v_0}{\kappa _1(1-\mu v_0)^3} + \frac{\lambda }{\kappa _1(1-\mu v_0)}\left( u_0-\frac{\mu v_0}{1-\mu v_0}\right) ^2\nonumber \\&\quad - \frac{\sigma }{\kappa _1}(\kappa _0+\kappa _1)\left( u_0 - \frac{\mu v_0}{1-\mu v_0}\right) \Bigg ], \end{aligned}$$

where

B.18 $$\begin{aligned} k_{0,M}&= \chi _1 (1+u_0)^{m_0}(1+v_0)^{n_0}, \end{aligned}$$

B.19 $$\begin{aligned} k_{1,M}&= \rho _M (1-\chi _0) m_0 (1+u_0)^{m_0-1}(1+v_0)^{n_0}\frac{1}{(1-\mu v_0)^2}, \end{aligned}$$

B.20 $$\begin{aligned} l_{1,M}&= -\chi _1(\kappa _0+\kappa _1)(1+u_0)^{m_0}(1+v_0)^{n_0} \nonumber \\&\quad - \rho _M (1-\chi _0) m_0 (1+u_0)^{m_0-1}(1+v_0)^{n_0} \left( u_0 - \frac{\mu v_0}{1-\mu v_0}\right) , \end{aligned}$$

B.21 $$\begin{aligned} k_{2,M}&= \chi _1 (1+u_0)^{m_0}(1+v_0)^{n_0} \frac{\lambda \kappa _1}{(1-\mu v_0)^2}\left( u_0-\frac{\mu v_0}{1-\mu v_0}\right) \nonumber \\&\quad - \rho _M (1+u_0)^{m_0+1}(1+v_0)^{n_0} (1-\chi _0) \frac{\lambda }{(1-\mu v_0)^2}\nonumber \\&\quad + \rho _M (1+u_0)^{m_0-1}(1+v_0)^{n_0} m_0 \frac{\kappa _0}{(1-\mu v_0)^3} \nonumber \\&\quad + \rho _M m_0 (1+u_0)^{m_0-1}(1+v_0)^{n_0}(1-\chi _0) \frac{1}{(1-\mu v_0)^2} \nonumber \\&\quad \times \Bigg [\frac{(\lambda +3)\mu v_0}{(1-\mu v_0)^2} + \frac{\lambda }{1-\mu v_0} \left( u_0-\frac{\mu v_0}{1-\mu v_0}\right) \nonumber \\&\quad - \lambda \left( u_0-\frac{\mu v_0}{1-\mu v_0}\right) ^2\Bigg ],\quad \text {and} \end{aligned}$$

B.22 $$\begin{aligned} l_{2,M}&= \chi _1 (1+u_0)^{m_0}(1+v_0)^{n_0} \frac{\lambda \kappa _1}{1-\mu v_0} \left( u_0-\frac{\mu v_0}{1-\mu v_0}\right) \nonumber \\&\quad - \rho _M (1+u_0)^{m_0}(1+v_0)^{n_0}(1-\chi _0) \frac{\lambda }{1-\mu v_0} \left[ \left( u_0-\frac{\mu v_0}{1-\mu v_0}\right) - \frac{1}{1-\mu v_0}\right] \nonumber \\&\quad - \rho _M (1+u_0)^{m_0-1}(1+v_0)^{n_0}m_0(1-\chi _0)\frac{1}{1-\mu v_0}\nonumber \\&\quad \times \Bigg [\frac{\kappa _0+\kappa _1 -1}{1-\mu v_0} + \frac{1}{(1-\mu v_0)^2} - \frac{\lambda }{1-\mu v_0} \left( u_0-\frac{\mu v_0}{1-\mu v_0}\right) \nonumber \\&\quad + \lambda \left( u_0-\frac{\mu v_0}{1-\mu v_0}\right) ^2 \Bigg ]. \end{aligned}$$

### Protein autoactivation

For the case of protein autoactivation, we replace $\alpha _M (u+1)\left( \frac{\partial u}{\partial u_0}\right) ^{-1}\partial _{u_0} F^{(0)}_{m-1}$ in Eqs. (3.45) and (3.46) with

B.23 $$\begin{aligned} \alpha _P (v+1)\left( \frac{\partial v}{\partial v_0}\right) ^{-1}\left[ -\left( \frac{\partial u}{\partial v_0}\right) \left( \frac{\partial u}{\partial u_0}\right) ^{-1}\partial _{u_0} + \partial _{v_0}\right] F^{(0)}_{m-1}, \end{aligned}$$

which yields

B.24 $$\begin{aligned} F^{(0)}_{1,0}&= h_{0,P}, \end{aligned}$$

B.25 $$\begin{aligned} F^{(0)}_{1,1}&= h_{1,P} + j_{1,P}\sigma - \alpha _P \chi _0 m_0 \mu (1+u_0)^{m_0-1}(1+v_0)^{n_0+1} \nonumber \\&\quad \times \Bigg [\frac{\mathrm{e}^{-(1-\mu v_0)\sigma }}{(1-\mu v_0)^3} + \frac{\sigma ^2}{2}\left( u_0 - \frac{\mu v_0}{1-\mu v_0}\right) \Bigg ],\quad \text {and} \end{aligned}$$

B.26 $$\begin{aligned} F^{(0)}_{1,2}&= h_{2,P} + j_{2,P}\sigma \nonumber \\&\quad + \alpha _P n_0 (1+u_0)^{m_0}(1+v_0)^{n_0-1} \frac{\sigma ^2}{2} \left\{ [2 (\kappa _0+\kappa _1)\chi _0 - \kappa _1](1+v_0) + \chi _0\right\} \nonumber \\&\quad - \alpha _P m_0 (1+u_0)^{m_0-1}(1+v_0)^{n_0} \mu \frac{\mathrm{e}^{-(1-\mu v_0)\sigma }}{(1-\mu v_0)^4}\nonumber \\&\quad \times \left[ \kappa _1(1+v_0) + \chi _0 \frac{1+2 v_0+\mu v_0(5+4 v_0)}{1-\mu v_0}\right] \nonumber \\&\quad +\alpha _P m_0 (1+u_0)^{m_0-1}(1+v_0)^{n_0} \sigma \mu \frac{\mathrm{e}^{-(1-\mu v_0)\sigma }}{(1-\mu v_0)^3}\nonumber \\&\quad \times \left\{ -\chi _0 \frac{[1+(3+2 v_0)\mu ]v_0}{1-\mu v_0}+[(1-\chi _0)\kappa _1 - \chi _0 \kappa _0](1+v_0)\right\} \nonumber \\&\quad + \alpha _P m_0 (1+u_0)^{m_0-1}(1+v_0)^{n_0} \mu \frac{\sigma ^2}{2} \frac{1}{(1-\mu v_0)^2} \nonumber \\&\quad \times \left\{ -\chi _0(1+v_0)\frac{\mu v_0 \mathrm{e}^{-(1-\mu v_0)\sigma }}{1-\mu v_0} + (1+v_0)[2(\kappa _0+\kappa _1)\chi _0 - \kappa _1] \right. \nonumber \\&\quad \left. + \chi _0 \frac{1+\mu v_0^2}{1-\mu v_0}\right\} \nonumber \\&\quad + \alpha _P m_0 (1+u_0)^{m_0-1}(1+v_0)^{n_0} \mu \frac{\sigma ^3}{6}\left( u_0-\frac{\mu v_0}{1-\mu v_0}\right) \left\{ (1+v_0)\right. \nonumber \\&\quad \left. \times [3 \chi _0(\kappa _0+\kappa _1)-2 \kappa _1] + \chi _0(1-v_0)\right\} \nonumber \\&\quad - (1+u_0)^{m_0}(1+v_0)^{n_0} \chi _1 \left\{ \frac{\lambda \kappa _1 \mathrm{e}^{(1-\mu v_0)\sigma }}{(1-\mu v_0)^2}\left( u_0-\frac{\mu v_0}{1-\mu v_0}\right) \right. \nonumber \\&\quad \left. + \frac{\sigma ^2}{2} \left[ \frac{\lambda \kappa _1 \mu v_0}{1-\mu v_0} - (\kappa _0+\kappa _1)^2\right] \right\} . \end{aligned}$$

Hence, it follows that

B.27 $$\begin{aligned} F^{(1)}_{1,0}&= \left( \frac{1}{\kappa _1} j_{1,P} + \kappa _0 h_{0,P}\right) +\frac{\alpha _P \chi _0}{\kappa _1} (1+u_0)^{m_0-1}(1+v_0)^{n_0} \left\{ \frac{m_0 \mu (1+v_0)}{(1-\mu v_0)^2} \right. \nonumber \\&\quad \left. + n_0 \left[ \left( u_0 - \frac{\mu v_0}{1-\mu v_0}\right) +\frac{1}{1-\mu v_0}\right] \right\} \quad \text {and} \end{aligned}$$

B.28 $$\begin{aligned} F^{(1)}_{1,1}&= \frac{j_{2,P} + \kappa _0(h_{1,P} + j_{1,P} \sigma )}{\kappa _1} \end{aligned}$$

B.29 $$\begin{aligned}&\quad - \frac{\chi _1}{\kappa _1} (1+u_0)^{m_0}(1+v_0)^{n_0}\left[ \frac{\lambda \kappa _1 \mathrm{e}^{(1-\mu v_0)\sigma }}{1-\mu v_0} \left( u_0 - \frac{\mu v_0}{1-\mu v_0}\right) \right. \nonumber \\&\quad \left. + \sigma \left( \frac{\lambda \kappa _1 \mu v_0}{1-\mu v_0} - (\kappa _0+\kappa _1)^2\right) \right] \nonumber \\&\quad + \alpha _P n_0 (1+u_0)^{m_0}(1+v_0)^{n_0} \sigma \chi _0\frac{\kappa _0+\kappa _1}{\kappa _1}\nonumber \\&\quad + \alpha _P m_0 (1+u_0)^{m_0-1}(1+v_0)^{n_0+1} \frac{\mu \chi _0}{\kappa _1} \left[ \frac{\kappa _1 \mathrm{e}^{-(1-\mu v_0)\sigma }}{(1-\mu v_0)^3}+\frac{1+2 \mu v_0}{(1-\mu v_0)^4}\right. \nonumber \\&\quad \left. +\sigma \frac{\kappa _0+\kappa _1}{(1-\mu v_0)^2}+\kappa _1\frac{\sigma ^2}{2}\left( u_0-\frac{\mu v_0}{1-\mu v_0}\right) \right] , \end{aligned}$$

where

B.30 $$\begin{aligned} h_{0,P}&= \chi _1 (1+u_0)^{m_0}(1+v_0)^{n_0}, \end{aligned}$$

B.31 $$\begin{aligned} h_{1,P}&= \alpha _P \chi _0 m_0 \mu (1+u_0)^{m_0-1}(1+v_0)^{n_0+1}\frac{1}{(1-\mu v_0)^3}, \end{aligned}$$

B.32 $$\begin{aligned} j_{1,P}&= -\chi _1(\kappa _0+\kappa _1)(1+u_0)^{m_0}(1+v_0)^{n_0} \nonumber \\&\quad - \alpha _P \chi _0 (1+u_0)^{m_0}(1+v_0)^{n_0}\left[ n_0 + \mu m_0 \frac{1+v_0}{1+u_0} \frac{1}{(1-\mu v_0)^2}\right] , \end{aligned}$$

B.33 $$\begin{aligned} h_{2,P}&= \chi _1 (1+u_0)^{m_0}(1+v_0)^{n_0} \frac{\lambda \kappa _1}{(1-\mu v_0)^2}\left( u_0-\frac{\mu v_0}{1-\mu v_0}\right) \nonumber \\&\quad +\alpha _P m_0 (1+u_0)^{m_0-1}(1+v_0)^{n_0} \frac{\mu }{(1-\mu v_0)^4}\nonumber \\&\quad \times \left[ (1+v_0)\kappa _1 + \chi _0 \frac{1+2 v_0+\mu v_0(5+4 v_0)}{1-\mu v_0}\right] ,\quad \text {and} \end{aligned}$$

B.34 $$\begin{aligned} j_{2,P}&= \chi _1 (1+u_0)^{m_0}(1+v_0)^{n_0} \frac{\lambda \kappa _1}{1-\mu v_0} \left( u_0-\frac{\mu v_0}{1-\mu v_0}\right) \nonumber \\&\quad + \alpha _P \chi _0 \mu m_0 (1+u_0)^{m_0-1}(1+v_0)^{n_0+1} \frac{1}{(1-\mu v_0)^3}\nonumber \\&\quad \times \left[ 2-(\kappa _0 + \kappa _1) - \frac{3}{1-\mu v_0}\right] . \end{aligned}$$

### Protein autorepression

For the case of protein autorepression, we replace $\alpha _M (u+1)\left( \frac{\partial u}{\partial u_0}\right) ^{-1}\partial _{u_0} F^{(0)}_{m-1}$ in Eqs. (3.45) and (3.46) with

B.35 $$\begin{aligned} -\rho _P (v+1)\left( \frac{\partial v}{\partial v_0}\right) ^{-1}\left[ -\left( \frac{\partial u}{\partial v_0}\right) \left( \frac{\partial u}{\partial u_0}\right) ^{-1}\partial _{u_0} + \partial _{v_0}\right] F^{(1)}_{m-1}, \end{aligned}$$

which yields

B.36 $$\begin{aligned} F^{(0)}_{1,0}&= k_{0,P},\end{aligned}$$

B.37 $$\begin{aligned} F^{(0)}_{1,1}&= k_{1,P} + l_{1,P}\sigma + \rho _P (1-\chi _0) m_0 \mu (1+u_0)^{m_0-1}(1+v_0)^{n_0+1} \nonumber \\&\times \left[ \frac{\mathrm{e}^{-(1-\mu v_0)\sigma }}{(1-\mu v_0)^3} + \frac{\sigma ^2}{2}\left( u_0-\frac{\mu v_0}{1-\mu v_0}\right) \right] ,\quad \text {and} \end{aligned}$$

$$\begin{aligned} F^{(0)}_{1,2}&= k_{2,P} + l_{2,P}\sigma \nonumber \\&\quad + \rho _P (1-\chi _0) \lambda \mu (1+u_0)^{m_0}(1+v_0)^{n_0+1} \frac{1}{(1-\mu v_0)^3}\nonumber \\&\quad \times \left[ \mathrm{e}^{(1-\mu v_0)\sigma }\left( u_0 - \frac{\mu v_0}{1-\mu v_0}\right) - \frac{\mathrm{e}^{-(1-\mu v_0)\sigma }}{1-\mu v_0}\right] \nonumber \\&\quad + \rho _P (1-\chi _0) \lambda n_0(1+u_0)^{m_0}(1+v_0)^{n_0} \mathrm{e}^{(1-\mu v_0)\sigma }(1-\mu v_0) \left( u_0 - \frac{\mu v_0}{1-\mu v_0}\right) \nonumber \\&\quad + \rho _P m_0 (1+u_0)^{m_0-1}(1+v_0)^{n_0+1} \frac{1}{(1-\mu v_0)^3} \frac{\kappa _0 \mu \mathrm{e}^{-(1-\mu v_0)\sigma }}{1-\mu v_0}\nonumber \\&\quad + \rho _P (1-\chi _0)m_0 (1+u_0)^{m_0-1}(1+v_0)^{n_0+1}\frac{\lambda \mu \mathrm{e}^{(1-\mu v_0)\sigma }}{(1-\mu v_0)^3}\left( u_0-\frac{\mu v_0}{1-\mu v_0}\right) \nonumber \\&\quad \times \left[ \frac{1}{1-\mu v_0} - \left( u_0-\frac{\mu v_0}{1-\mu v_0}\right) \right] \nonumber \\&\quad + \rho _P m_0 (1+u_0)^{m_0-1}(1+v_0)^{n_0+1} \frac{\mu \mathrm{e}^{-(1-\mu v_0)\sigma }}{(1-\mu v_0)^4}\nonumber \\&\quad \times \left[ -\lambda \left( u_0 - \frac{\mu v_0}{1-\mu v_0}\right) + \frac{6}{1-\mu v_0} + \frac{\lambda \mu v_0}{1-\mu v_0} - \frac{5 + 4 v_0}{1+v_0}\right] \nonumber \\&\quad + \rho _P \sigma \,\lambda \mu m_0 (1-\chi _0) (1+u_0)^{m_0-1}(1+v_0){n_0+1} \frac{\mathrm{e}^{(1-\mu v_0)\sigma }}{(1-\mu v_0)^2}\nonumber \\&\quad \times \left( u_0-\frac{\mu v_0}{1-\mu v_0}\right) ^2 \nonumber \\&\quad + \rho _P \sigma \,\mu m_0 (1+u_0)^{m_0-1}(1+v_0){n_0}\frac{\mathrm{e}^{-(1-\mu v_0)\sigma }}{(1-\mu v_0)^3} \Bigg [\chi _0 (1+v_0)(\kappa _0+\kappa _1) \nonumber \\&\quad -(1+v_0)\kappa _1+ (1-\chi _0)\frac{v0 + \mu v_0(3+2 v_0) + \mu v_0 \lambda (1+v_0)}{1-\mu v_0}\Bigg ]\nonumber \\&\quad + \rho _P \frac{\sigma ^2}{2} (1+u_0)^{m_0}(1+v_0)^{n_0+1} (1-\chi _0) \frac{\lambda \mu }{1-\mu v_0} \nonumber \\&\quad \times \left[ \frac{1}{1-\mu v_0} - \left( u_0 - \frac{\mu v_0}{1-\mu v_0}\right) \right] \nonumber \\&\quad + \rho _P \frac{\sigma ^2}{2} n_0 (1+u_0)^{m_0}(1+v_0)^{n_0}\left[ -(1-\chi _0)\left( \frac{1}{1+v_0} + \frac{\lambda \mu v_0}{1-\mu v_0}\right) \right. \nonumber \\&\quad \left. -(1-2 \chi _0)(\kappa _0+\kappa _1) -\kappa _1\right] + \rho _P \frac{\sigma ^2}{2} \mu m_0 (1+u_0)^{m_0-1}(1+v_0)^{n_0+1}\nonumber \\&\quad \times (1-\chi _0) \frac{\mu v_0 \mathrm{e}^{-(1-\mu v_0)\sigma }}{(1-\mu v_0)^3}\nonumber \\&\quad + \rho _P \frac{\sigma ^2}{2} \mu m_0 (1+u_0)^{m_0-1}(1+v_0)^{n_0} \Bigg \{[-(1-2\chi _0)(\kappa _0+\kappa _1)-\kappa _1] \nonumber \\ \end{aligned}$$

B.38 $$\begin{aligned}&\frac{1+v_0}{(1-\mu v_0)^2}- (1-\chi _0)\frac{1+\mu v_0^2}{(1-\mu v_0)^3} + (1-\chi _0)(1+v_0) \nonumber \\&\times \left[ \frac{\mu v_0}{(1-\mu v_0)^3} + \frac{1}{1-\mu v_0} \left( u_0 - \frac{\mu v_0}{1-\mu v_0}\right) ^2\right] \Bigg \} \nonumber \\&+ \rho _P \frac{\sigma ^3}{6} \mu m_0 (1+u_0)^{m_0-1}(1+v_0)^{n_0} \left( u_0-\frac{\mu v_0}{1-\mu v_0}\right) \nonumber \\&\times \Bigg [(-(1-3\chi _0)(\kappa _0+\kappa _1)-2 \kappa _1)(1+v_0) \nonumber \\&+(1-\chi _0) \left( v_0-1 + 2 \lambda \frac{\mu v_0(1+v_0)}{1-\mu v_0}\right) \Bigg ]\nonumber \\&- (1+u_0)^{m_0}(1+v_0)^{n_0} \chi _1 \left[ \frac{\lambda \kappa _1 \mathrm{e}^{(1-\mu v_0)\sigma }}{(1-\mu v_0)^2} \left( u_0 - \frac{\mu v_0}{1-\mu v_0}\right) \right. \nonumber \\&\times \left. + \frac{\sigma ^2}{2}\left( \frac{\lambda \kappa _1 \mu v_0}{1-\mu v_0}-(\kappa _0+\kappa _1)^2\right) \right] . \end{aligned}$$

It follows that

B.39 $$\begin{aligned} F^{(1)}_{1,0}&= \frac{1}{\kappa _1} \left( l_{1,P} + \kappa _0 k_{0,P}\right) \nonumber \\&\quad -\rho _P m_0 (1-\chi _0) (1+u_0)^{m_0-1}(1+v_0)^{n_0}\frac{1}{\kappa _1}\nonumber \\&\quad \times \left\{ \frac{m_0 \mu (1+v_0)}{(1-\mu v_0)^2} +n_0\left[ \frac{1}{1-\mu v_0}+ \left( u_0 - \frac{\mu v_0}{1-\mu v_0}\right) \right] \right\} \quad \text {and} \end{aligned}$$

B.40 $$\begin{aligned} F^{(1)}_{1,1}&= \frac{l_{2,M} + \kappa _0(k_{1,M} + l_{1,M} \sigma )}{\kappa _1} \nonumber \\&\quad + \rho _P (1-\chi _0)(1+u_0)^{m_0+1}(1+v_0)^{n_0+1} \frac{\lambda \mu }{\kappa _1} \frac{1}{(1-\mu v_0)^2}\nonumber \\&\quad + \rho _P n_0 (1-\chi _0)(1+u_0)^{m_0}(1+v_0)^{n_0} \frac{1}{\kappa _1}\nonumber \\&\quad \times \left[ \frac{\lambda }{1-\mu v_0}\left( u_0-\frac{\mu v_0}{1-\mu v_0}\right) -(\kappa _0+\kappa _1)\sigma \right] \nonumber \\&\quad + \rho _P\,\mu m_0 (1-\chi _0)(1+u_0)^{m_0-1}(1+v_0)^{n_0+1}\frac{1}{\kappa _1}\nonumber \\&\quad \times \Bigg \{-\frac{3}{(1-\mu v_0)^4} +\frac{1}{(1-\mu v_0)^3}\left[ -\kappa _1 \mathrm{e}^{-(1-\mu v_0)\sigma }+2 \right. \nonumber \\&\quad \left. + 2 \lambda \left( u_0-\frac{\mu v_0}{1-\mu v_0}\right) \right] -\sigma \frac{\kappa _0+\kappa _1}{(1-\mu v_0)^2}-\frac{\sigma ^2}{2} \kappa _1 \left( u_0-\frac{\mu v_0}{1-\mu v_0}\right) \Bigg \}\nonumber \\&\quad - \frac{\chi _1}{\kappa _1} (1+u_0)^{m_0}(1+v_0)^{n_0}\nonumber \\&\quad \times \left[ \frac{\lambda \kappa _1 \mathrm{e}^{(1-\mu v_0)\sigma }}{1-\mu v_0} \left( u_0 - \frac{\mu v_0}{1-\mu v_0}\right) + \sigma \left( \frac{\lambda \kappa _1 \mu v_0}{1-\mu v_0} - (\kappa _0+\kappa _1)^2\right) \right] , \end{aligned}$$

where

B.41 $$\begin{aligned} k_{0,P}&= \chi _1 (1+u_0)^{m_0}(1+v_0)^{n_0}, \end{aligned}$$

B.42 $$\begin{aligned} k_{1,P}&= -\rho _P (1-\chi _0) m_0 (1+u_0)^{m_0-1}(1+v_0)^{n_0+1}\frac{\mu }{(1-\mu v_0)^3}, \end{aligned}$$

B.43 $$\begin{aligned} l_{1,P}&= -\chi _1(\kappa _0+\kappa _1)(1+u_0)^{m_0}(1+v_0)^{n_0}\nonumber \\&\quad - \rho _P (1-\chi _0) (1+u_0)^{m_0}(1+v_0)^{n_0}\left[ n_0 + \mu m_0 \frac{1+v_0}{1+u_0} \frac{1}{(1-\mu v_0)^2}\right] , \end{aligned}$$

B.44 $$\begin{aligned} k_{2,P}&= \chi _1 (1+u_0)^{m_0}(1+v_0)^{n_0} \frac{\lambda \kappa _1}{(1-\mu v_0)^2}\left( u_0-\frac{\mu v_0}{1-\mu v_0}\right) \nonumber \\&\quad + \rho _P(1-\chi _0) (1+u_0)^{m_0}(1+v_0)^{n_0+1} \frac{\lambda \mu }{(1-\mu v_0)^3} \nonumber \\&\quad \times \left[ \frac{1}{1-\mu v_0}-\left( u_0-\frac{\mu v_0}{1-\mu v_0}\right) \right] \nonumber \\&\quad - \rho _P (1-\chi _0) n_0 (1+u_0)^{m_0}(1+v_0)^{n_0} \frac{\lambda }{(1-\mu v_0)^2}\left( u_0-\frac{\mu v_0}{1-\mu v_0}\right) \nonumber \\&\quad + \rho _P (1-\chi _0) m_0 \mu (1+u_0)^{m_0-1}(1+v_0)^{n_0+1} \frac{\lambda }{(1-\mu v_0)^3}\nonumber \\&\quad \times \left[ \left( u_0-\frac{\mu v_0}{1-\mu v_0}\right) ^2 - \frac{\mu v_0}{(1-\mu v_0)^2}\right] \nonumber \\&\quad - \rho _P m_0 (1+u_0)^{m_0-1}(1+v_0)^{n_0} \frac{\mu }{(1-\mu v_0)^4}\nonumber \\&\quad \times \left\{ \frac{1-\chi _0}{1-\mu v_0}[1+2 v_0+\mu v_0(5+4 v_0)]+(1+v_0)\kappa _0\right\} ,\quad \text {and} \end{aligned}$$

B.45 $$\begin{aligned} l_{2,P}&= \chi _1 (1+u_0)^{m_0}(1+v_0)^{n_0} \frac{\lambda \kappa _1}{1-\mu v_0} \left( u_0-\frac{\mu v_0}{1-\mu v_0}\right) \nonumber \\&\quad - \rho _P (1+u_0)^{m_0+1}(1+v_0)^{n_0+1}(1-\chi _0) \frac{\lambda \mu }{(1-\mu v_0)^2}\nonumber \\&\quad - \rho _P n_0(1+u_0)^{m_0}(1+v_0)^{n_0}(1-\chi _0) \frac{\lambda }{1-\mu v_0}\left( u_0-\frac{\mu v_0}{1-\mu v_0}\right) \nonumber \\&\quad + \rho _P \mu m_0 (1+u_0)^{m_0-1}(1+v_0)^{n_0+1} (1-\chi _0) \frac{1}{(1-\mu v_0)^3}\nonumber \\&\quad \times \left[ -2-2 \lambda \left( u_0-\frac{\mu v_0}{1-\mu v_0}\right) +\frac{3}{1-\mu v_0}+\kappa _0+\kappa _1\right] . \end{aligned}$$

## Footnotes

1 In some specific cases, however, such explicit expressions can be found; see e.g. Popović et al. (2016).
